# Western Diet Induces Impairment of Liver-Brain Axis Accelerating Neuroinflammation and Amyloid Pathology in Alzheimer's Disease

**DOI:** 10.3389/fnagi.2021.654509

**Published:** 2021-04-01

**Authors:** Angelika Wiȩckowska-Gacek, Anna Mietelska-Porowska, Dominik Chutorański, Małgorzata Wydrych, Jan Długosz, Urszula Wojda

**Affiliations:** Laboratory of Preclinical Testing of Higher Standard, Neurobiology Center, Nencki Institute of Experimental Biology, Polish Academy of Sciences, Warsaw, Poland

**Keywords:** Alzheimer's disease, western diet, metabolic syndrome, liver-brain axis, neuroinflammation, astroglia, microglia, amyloid

## Abstract

Alzheimer's disease (AD) is an aging-dependent, irreversible neurodegenerative disorder and the most common cause of dementia. The prevailing AD hypothesis points to the central role of altered cleavage of amyloid precursor protein (APP) and formation of toxic amyloid-β (Aβ) deposits in the brain. The lack of efficient AD treatments stems from incomplete knowledge on AD causes and environmental risk factors. The role of lifestyle factors, including diet, in neurological diseases is now beginning to attract considerable attention. One of them is western diet (WD), which can lead to many serious diseases that develop with age. The aim of the study was to investigate whether WD-derived systemic disturbances may accelerate the brain neuroinflammation and amyloidogenesis at the early stages of AD development. To verify this hypothesis, transgenic mice expressing human APP with AD-causing mutations (APPswe) were fed with WD from the 3rd month of age. These mice were compared to APPswe mice, in which short-term high-grade inflammation was induced by injection of lipopolysaccharide (LPS) and to untreated APPswe mice. All experimental subgroups of animals were subsequently analyzed at 4-, 8-, and 12-months of age. APPswe mice at 4- and 8-months-old represent earlier pre-plaque stages of AD, while 12-month-old animals represent later stages of AD, with visible amyloid pathology. Already short time of WD feeding induced in 4-month-old animals such brain neuroinflammation events as enhanced astrogliosis, to a level comparable to that induced by the administration of pro-inflammatory LPS, and microglia activation in 8-month-old mice. Also, WD feeding accelerated increased Aβ production, observed already in 8-month-old animals. These brain changes corresponded to diet-induced metabolic disorders, including increased cholesterol level in 4-months of age, and advanced hypercholesterolemia and fatty liver disease in 8-month-old mice. These results indicate that the westernized pattern of nourishment is an important modifiable risk factor of AD development, and that a healthy, balanced, diet may be one of the most efficient AD prevention methods.

## Introduction

Currently progress in scientific research has resulted in an increase in life expectancy; unnatural for our species compared to previous centuries. The consequences of this phenomenon are age-related diseases: an increasingly important public health and economic challenge in the world. The most commonly diagnosed age-dependent neurological disorder is Alzheimer's disease (AD) (Ryu et al., 2019). According to the Alzheimer's Association report 1 in 10 people who are older than 65, and 1 in 2 over 85 will suffer from AD (Alzheimer's Association, 2020). The World Health Organization (World Health Organization, 2019) and the Alzheimer's Association predict that by 2050 the number of AD patients will triple. Simultaneously, the last 2–3 generations have experienced the epidemics of obesity and non-infectious degenerative diseases, widely understood as “civilization diseases” (World Health Organization, 2019). This has culminated in the most common causes of death becoming disorders, such as cancer, cardiovascular diseases, diabetic complications (Kopp, 2019), and neurodegenerative diseases. All of these disorders are related to metabolic impairment, which is increasingly focusing the attention of scientists and physicians on the role of lifestyle factors, including nutrition, in the development of these diseases.

AD was first described at the beginning of the 20th century, and since then, intensive research has been carried out to understand the causes of its development and molecular mechanisms. Today we know that AD is a multiform disease with a very complex etiology and a long, latent, preclinical phase. Due to these features, despite the considerable progress in knowledge, we still cannot effectively treat patients suffering from AD (Long and Holtzman, 2019). The two most typical neuropathological changes observed in the brains of AD patients are deposits of amyloid-β (Aβ) peptide accumulating predominantly in the extracellular space in the form of senile plaques and in brain blood vessels as cerebral amyloid angiopathy (CAA), and deposits of over-phosphorylated forms of tau protein forming intracellular neurofibrillary tangles (Dubois et al., 2016). According to the prevailing amyloid cascade hypothesis, the first phenomenon in the progression of AD is appearance of the pathological Aβ peptides, a product of the amyloidogenic proteolysis of amyloid precursor protein (APP) by the β-secretase and γ-secretase transmembrane protein complexes. This process is enhanced by mutations in genes encoding APP or one of proteins constituting an enzymatic core of γ-secretase—presenilin 1 or presenilin 2. Such mutations cause development of hereditary, early onset, familial form of AD (FAD). FAD accounts for about 1% of diagnosed AD incidences, while the biggest problem is sporadic, late onset AD (SAD), occurring in close to 99% AD incidences (Cheng et al., 2020). Many hypotheses have arisen to define causes, molecular mechanisms and their sequence in the development of SAD, however, none of them covers the complexity of the etiology of this form of AD (Du et al., 2018). Most of the currently known potential risk factors for SAD, i.e., inflammation, hypertension, insulin resistance, type 2 diabetes mellitus, cardiovascular diseases, past infections and others, support the new hypothesis that environmental and modifiable factors related to lifestyle can occupy the dominant role in the molecular mechanisms responsible for development of AD (Edwards et al., 2019). Inadequate diet can lead to the development of metabolic syndrome (MetS), which includes many of the above known risk factors for AD. An unhealthy diet, through MetS development, has a negative effect on the whole body beginning in early childhood, and gradually impairing the proper functioning of the nervous system (Ricci et al., 2017). The WHO considers modifiable risk factors, such as diet, a significant aspect of AD prevention (World Health Organization, 2019). However, while animal studies have demonstrated the harmful effect of disbalanced content of particular diet macroelements (fat, sugar, cholesterol) on factors including memory, cognition, amyloidogenic accumulation of toxic Aβ peptides or neuroinflammation (Julien et al., 2010; Hohsfield et al., 2014; Vandal et al., 2014; Graham et al., 2016; Yeh et al., 2020), the sequence of pathological events and molecular mechanisms by which deficient diets like the western diet and related peripheral metabolic disturbances affect brain pathology have not been fully elucidated yet.

The western type of nourishment, referred to as western diet (WD), is known as one of the biggest risk factors to health and significantly contributes to the development of MetS. This originated in the USA, but is spreading very quickly among many developing countries in the world (Popkin et al., 2012; Kopp, 2019). Based on ultra-processed foods ready for consumption low in nutritional value (Monteiro et al., 2013), WD is characterized by a high content of simple sugars to the disadvantage of complex carbohydrates, a high content of salt, saturated fatty acids, trans fat acids and cholesterol. It also includes low content of grain, fiber and mono- and polyunsaturated fatty acids, including anti-inflammatory omega-3, -6, and -9 acids, vitamins and microelements. WD causes such impairments as obesity, type 2 diabetes mellitus, hyperinsulinemia and insulin resistance, hypercholesterolemia (HChol), non-alcoholic fatty liver disease (NAFLD), non-alcoholic steatohepatitis (NASH), and systemic inflammation collectively known as MetS (Luque-Contreras et al., 2014; Hosseini-Esfahani et al., 2018; Dabke et al., 2019). MetS is known to lead to atherosclerosis, coronary artery disease, strokes, hypertension, autoimmune disease, osteoporosis, and cancer (Kopp, 2019). There are significant links between WD-induced MetS and development of AD (Solfrizzi et al., 2004; Whitmer et al., 2008; Gustafson et al., 2009, 2012; Besser et al., 2014). It seems that diet-derived MetS may enhance neurodegeneration mainly by causing impairment in the blood brain barrier (BBB) resulting in enhanced accumulation of Aβ in the brain and triggering neuroinflammation.

The formation and deposition of Aβ peptides is a process that also occurs in the healthy brain and does not necessarily result in cognitive impairment (Rodrigue et al., 2012; Bassendine et al., 2020). Progression to AD then, is determined by the imbalance between the processes of Aβ formation and its degradation and effective removal from the brain. The brain's mechanisms of Aβ removal and degradation include: (1) phagocytosis and endocytosis by perivascular macrophages, microglia, and astrocytes and (2) proteolytic degradation by enzymes, such as insulin-degrading enzyme, neprilysin, and matrix metalloproteinases (Xiang et al., 2015). Equally important in cleansing Aβ from the brain and body are the peripheral organs and tissues, among which the blood and the liver seem to play the major roles (Cheng et al., 2020).

The main functions of the liver in a healthy organism include lipid metabolism, immunomodulation, detoxification, and endocrine activity (Cheng et al., 2020). Due to its detoxifying function, the liver is an organ that degrades defective and dangerous products, including circulating Aβ peptides. The significant role of the liver in the clearance of Aβ is based on the high affinity of Aβ for the circulating high-density lipoprotein (HDL) with which Aβ is delivered from the periphery to the liver (Robert et al., 2017). 70–90% of the plasma Aβ content is transported to the liver via the soluble plasma form of low-density lipoprotein receptor-related protein 1 (sLRP1). High LRP1 expression was also confirmed in hepatocytes, where Aβ is degraded with the participation of the bile. Biliary clearance of Aβ is not only mediated by LRP1, but also by the drug efflux pump: P-glycoprotein. Interestingly, the LRP1 in the liver is very susceptible to changes in response to pathological processes including inflammatory reactions, and can be quickly and easily lowered, leading to impairment of liver effectiveness in Aβ removal, followed by Aβ accumulation, both in the vessels (CAA) and in the brain (Tamaki et al., 2006; Sagare et al., 2007; Mohamed and Kaddoumi, 2013).

The balance between Aβ efflux from the brain to the periphery and its influx from the periphery into the brain is tightly controlled at the level of the BBB (Deane et al., 2004; Hartz et al., 2010; Ito et al., 2014). While the passage of Aβ peptides from the circulation into the brain across the BBB is mediated mainly by the multi-ligand receptor for advanced glycation end products (RAGE) (Montagne et al., 2017), the LRP1 receptors are responsible for the removal of toxic Aβ from the brain into circulation. The imbalance of these two processes from increased expression of RAGE can lead to the excessive accumulation of Aβ in the brain parenchyma in the form of Aβ plaques (Mietelska-Porowska and Wojda, 2017; Sweeney et al., 2019). The BBB's permeability impairment in AD development also causes increased infiltration of circulating immune cells from the periphery into the central nervous system. This phenomenon together with deposition of Aβ plaques can strongly contribute to neuroinflammation and gradual disturbance of neuronal functions.

Extensive data show that neuroinflammation plays a crucial role in development of AD (Schwabe et al., 2020). Neuroinflammation manifests in the brain as a strong activation of inflammatory systems comprising pro-inflammatory cytokines, chemokines, and glial cells; both microglia and astrocytes (Kaur et al., 2019; Streit et al., 2020). According to the classical hypothesis, Aβ deposition in the brain activates microglia. Activated microglia have been proposed to recruit astrocytes that actively enhance the brain inflammatory response to Aβ (Lian et al., 2016). This leads to a local, cytokine-mediated, acute-phase response, activation of the complement cascade and induction of pro-inflammatory response. All of these factors can contribute to neuronal dysfunction and death, either alone or in concert (Bezzi et al., 2001a,b; Abbas et al., 2002; Brown and Bal-Price, 2003; Allendorf et al., 2020; Yuan et al., 2020). Thus, the switch of microglia to pro-inflammatory polarization appears crucial for progression of neurodegeneration.

In recent years the number of publications documenting the role of diet and related metabolic alterations in brain impairment has significantly increased. The hypothesis tested in our study was based on the collection of available knowledge on: (1) the impact of individual dietary components on brain function, (2) the impact of WD on the development of metabolic syndrome, including NAFLD, (3) the role of WD as a pro-inflammatory factor, (4) inflammation-dependent and metabolic disturbance-dependent damage to the structure and function of the BBB in AD, (5) the role of the BBB in amyloid-β distribution, and the molecular components involved in amyloid-β transport through the BBB, and (6) the neuropathological characteristics of AD. Based on these premises, we hypothesized that WD, by inducing peripheral metabolic disturbances including NAFLD and a low-grade inflammatory state, accelerates AD pathogenesis, particularly development of neuroinflammation and amyloid pathology.

To verify the above presented hypothesis we used transgenic mice expressing human APP with AD-causing mutations (APPswe) fed with WD from the 3rd month of age, and for the first time we tracked and characterized the detailed chronology of WD-induced systemic and metabolic alterations followed by brain Aβ pathology and neuroinflammatory changes. We found that WD induced liver-brain-axis impairment and accelerated onset of brain neuroinflammation and amyloidogenesis in the early stages of AD development.

## Materials and Methods

### Animals, Study Groups, and Timeline of Experimental Procedures

Ten-week-old, male, transgenic APPswe mice [Tg line #1349; B6; SJL-Tg(APPSWE)2576Kha], were purchased from TACONIC BIOSCIENCES Laboratory, USA. APPswe mice carry a transgene coding for the 695-amino acid isoform of human APP with the Swedish mutation (APP_695_ gene containing the double mutation K670N, M671L) and develop amyloid plaques in the brain at a late age. All animal experimentation was performed in accordance with institutional guidelines following approvals by the First Ethical Committee in Warsaw (no 783/2015, 24/2015 217/2016, 630/2018) and in accordance with the Act of 15 Jan 2015 on the protection of animals used for scientific or educational purposes and Directive 2010/63/EU of the European Parliament and of the Council of 22 Sep 2010 on the protection of animals used for scientific purposes. Upon delivery mice were maintained in individually ventilated cages (IVC, Techniplast, Italy), light/dark 12/12 h, 22 (±2)°C temperature and 40–60% humidity conditions and provided with water and dry food pellets *ad libitum*.

All experiments were started in 13-week-old animals and continued until 20-month of age. Four mice experimental groups were created by a random assignment of animals: (1) control (CTR) group was fed with the standard chow-diet during the whole experiment, i.e., until 20 months; (2) LPS group was injected intraperitoneally with LPS at the beginning of the experiment, i.e., in 13-week-old animals (in accordance with the protocol described below), and fed for the whole experiment with the control chow-diet; (3) WD group was fed with western diet from the beginning until the end of the experiment (WD feeding started in 13-week-old animals and ended at 20-month-old); and (4) WD + LPS—group fed with WD during the whole experiment, like the WD group, and also injected with LPS at the beginning of the experiment, like the LPS group.

During the course of the experiment, five age subgroups of mice were created: 4-, 8-, 12-, 16-, and 20-month-old (4M, 8M, 12M, 16M, and 20M). Unfortunately, due to high mortality, we were unable to keep all mice alive in 16M and 20M experimental groups. Therefore, the quantitative analyses were performed in 4M, 8M, and 12M mice in all experimental groups: CTR, LPS, WD, and WD + LPS. The number of animals in a particular experimental and age group is given in Table 1. Data from 16M and 20M mouse groups are presented as enriching information obtained from qualitative analyses of incomplete groups.

**Table 1:** Number of animals per group.

	**4-month-old (4M)**	**8-month-old (8M)**	**12-month-old (12M)**
CTR	*n* = 7	*n* = 8	*n* = 6
LPS	*n* = 4	*n* = 4	*n* = 4
WD	*n* = 4	*n* = 5	*n* = 5
WD + LPS	*n* = 5	*n* = 5	*n* = 5

### The Induction of Systemic Inflammation by Lipopolysaccharide Administration

Thirteen-week-old mice were intraperitoneally injected with LPS at a dose of 0.5 mg/kg bm on 3 consecutive days. The LPS dose and experiment paradigm was determined as described earlier (Cazareth et al., 2014). The aim of LPS administration was to induce acute, high-grade, systemic inflammation (Gasparotto et al., 2017) which may cause a neuroinflammatory reaction in the brain (Lopes, 2016) and may influence the expression and processing of APP leading to generation of Aβ (Sheng et al., 2003).

Because the systemic and cerebral response to LPS administration is well-characterized, it was expected that LPS (used once only at starting the experiment) would cause a strong and short-term increase of neuroinflammatory markers. Thus, LPS was used in this study as a reference, to test activation of neuroinflammation. We tested if and when WD-induced changes would produce an effect similar to or comparable to that observed in young animals after acute administration of LPS.

### Composition of Western Diet and Balanced Control Diet

Tables 2, 3 show detailed comparison of both diets applied in this study: balanced normal control diet (R/M-H V1534) and western diet (EF R/M E15126-34), both sourced from Sniff® Company. Western diet is characterized by high levels of saturated fatty acids, simple carbohydrates and cholesterol.

**Table 2:** Percentage composition of diet ingredients.

**Nutrients**	**Control chow-diet [%]**	**Western diet [%]**
Dry matter	87.7	96.6
Crude protein	19.0	20.7
Crude fat	**3.3**	**30.0**
Crude fiber	4.9	5.0
Crude ash	6.4	5.6
N free extracts	54.1	34.3
Starch	**36.5**	**17.2**
Sugar	**4.7**	**16.3**
Fatty acids	3.25	26.87
Saturated fatty acids	**0.57**	**14.19**
Unsaturated fatty acids	**2.68**	**12.68**
Cholesterol	–	**284 [mg/kg]**

*Bold values are intended to emphasize the content of components of the WD and SD diets differ from each other. Highlighted values of dietary ingredients are of particular importance in the context of the described changes*.

**Table 3:** Percentage composition of energy derived from particular diet ingredients.

**Nutrients**	**Control chow-diet [%]**	**Western diet [%]**
Carbohydrates	58.0	29.0
Fat	9.0	54.0
Protein	33.0	17.0

### Mouse Euthanasia and Brain Tissue Collection

Animals had last free access to food 3 h before the terminal anesthesia. Prior to performing an intraperitoneal injection with a mixture of medetomidine at a dose of 1 mg/kg (ORION, Dexdomitor 0.5 mg/ml) and ketamine at a dose 75 mg/kg (Biowet, Ketamine 100 mg/ml) in sterile 0.9% NaCl, mice were sedated with isoflurane vapors (Baxter, AErrane 100% Liquid Inhalation Vapor) for 30 s to minimize the stress associated with entering the necropsy room and performing injection.

After euthanasia and transcardial perfusion with cold PBS supplemented with 0.1% sodium orthovanadate (Sigma Aldrich) and 1% heparin, mouse brains and internal organs were collected on ice. All brains were divided into two hemispheres. Right hemispheres were fixed in 10% buffered formalin and processed to prepare paraffin sections for histological and immunohistochemical analysis. From the brain left hemispheres, four fragments were separated for tissue lysate preparation and immunoblotting: hippocampus, entorhinal cortex, frontal cortex, and occipital cortex. The tissue lysates were first rapidly frozen in liquid nitrogen, and then stored at −80°C until the day of homogenate preparation for biochemical analysis. Right hemispheres of the mouse brains were fixed in 40% formalin and processed to prepare paraffin sections for histological and immunohistochemical analysis.

### Human Brain Tissue Collection

Human brain tissue was obtained from the collection of the Polish Brain Bank in the Institute of Psychiatry and Neurology. The archival material was preserved in a 10% buffered formalin. Tissue fragments were automatically processed in a fully enclosed system (Leica HistoCore Pearl Tissue Processor). The tissue samples were rinsed with distilled water for 24 h and then dehydrated in an increasing alcohol series (6 changes). The material was then cleared in xylene (4 changes). The tissue was saturated with liquid paraffin (62°C) in three changes for 2 h each and then embedded in blocks. Paraffin blocks were cut into slices 8 μm thick.

### Mouse Blood Collection

Blood was drawn straight from the heart of an anesthetized mouse immediately before perfusion into SARSTEDT test tubes with Li-heparin or EDTA K for biochemical and hematological analysis, respectively. Plasma for biochemical analysis was obtained from collected blood samples by centrifugation at 4,065 rcf for 30 min at 15°C.

### Plasma Total Cholesterol Concentration

Plasma cholesterol concentration was determined using a biochemical analyzer working in dry-plate technology (Fuji DriChem NX500 Automated Clinical Chemistry Analyzer).

### Hematological Analysis

Hematological analysis was performed using an automatic hematology analyzer (SCIL Vet ABC Hematology Analyzer, Scil Animal Care Company GmbH) employing an electrical impedance technique.

### Tissue Preparation for Immunohistochemical Staining

Tissues of the mouse brain and internal organs were fixed with 10% buffered formalin for 14 days and then dehydrated in 70% EtOH prior to automatic processing and paraffinization in an automatic tissue processor (Spin Tissue STP 120, Thermo Scientific) in a series of increasing concentrations of EtOH, three changes of xylene and two changes of liquid paraffin at 60°C. In order to prepare the paraffin blocks, the processed tissue fragments were embedded using an embedding workstation (HistoStar, Thermo Scientific). Paraffin blocks were cut on a rotary microtome (Thermo Scientific): blocks from the brain hemisphere into 8 μm thick slices and from the livers into 5 μm thick slices.

### Immunofluorescence (GFAP, Iba1, Aβ)

Before immunostaining with specific antibodies, the deparaffinized and hydrated tissue slices were subjected to the antigen retrieval process by incubation with 10 mM sodium citrate buffer (pH 6) with 0.05% Tween20, in three 5 min heating sessions in a microwave (600 V), separated by gradual cooling first at room temperature (RT) and then on ice. After the revealing of epitopes, tissue was washed with saline with 0.3% Triton X-100 (PBS-TX) and placed for 2 h in phosphate buffer blocking/permeabilizing solution containing 5% normal goat serum and 1% BSA in PBS-TX. Next, brain slices were incubated for 2 h at RT, and next for 16 h at 4°C with one of the following antibodies: rabbit anti-GFAP (ab7260), rabbit anti-Iba1 [EPR16588], (ab178846) or mouse anti-β-Amyloid, 1–16 [6E10] (BioLegend 803001). Then tissue slices were washed with PBS-TX (3 times for 5 min) and incubated for 2 h at RT with the respective Alexa Fluor conjugated antibody (ab150077, ab150116, ab150080), protected from light. Finally, tissue slices were washed, incubated for 10 min with Hoechst (Sigma Aldrich) to visualize nuclei, washed again with PBS-TX, and mounted with Mowiol 4-88 (Sigma Aldrich).

### Histology—Liver Staining

Histological structure visualization of liver tissues were done by routine topographic staining with hematoxylin and eosin (H&E). The liver tissue slides were stained using an automatic stainer (Thermo Scientific, Varistain Gemini ES Automated Slide Stainer) following a standard protocol. First, 5 μm thick liver slices were deparaffinized and rehydrated by serial incubations with xylene and alcohol mixtures of different percentages. Then, the tissue was stained for 2 min in Mayer's Hematoxylin solution (O. Kindler &ORSAtec), differentiated in acid alcohol (1% HCl) for 1 min, washed in bluing reagent (Shandon) for 4 min, and incubated for 7 min in 0.2% EOSIN Y alcoholic solution (O. Kindler &ORSAtec). All steps were separated by 1 min washes in distilled water. Next, the liver tissue slices were dehydrated using a number of washes in alcohol and xylene mixtures of different percentages (70, 96, 100% ethanol for 30 s each and 3 changes of xylene, 1 min each). Finally, the slides were manually mounted with the xylene based mounting medium (Consul-Mount, Thermo Scientific Shandon) and coverslipped.

### Image Collection and Analysis of Immunofluorescence

Immunofluorescence of immunostained brain slices was analyzed with fluorescent and light microscope Nikon Eclipse Ni. Brain tissue photos were taken with Image Pro-Plus 7 (Media Cybernetic) and NIS (Nikon) software under lens magnification × 10, × 20, and × 40 (Nikon, SLR camera lenses, CFI Plan Apochromat Lambda objectives, NA: 10×/0.30, 20×/0.50, 40×/0.75). Hippocampal sections were analyzed at the AP level within the range −2.00 and −2.50 from Bregma. Quantitative analysis of Iba1 positive staining area was performed using ImageJ v.1.46 and Image Pro-Plus 7 (Media Cybernetic) software. Immunofluorescence images (.tif) in grayscale from ×20 optical magnification were transformed into binary images with applied threshold, calculated by the software by cutting the tissue autofluorescence background and non-specific artifacts. Next, the quantity of Iba1 positively stained area was measured from different parts and cross-sections of the hippocampus (from 3 to 13 per animal) and presented as the mean percentage Iba1 positive area (in μm^2^) always measured in equal hippocampus tissue area (region of interest: ROI = 78525.65 μm^2^).

### SDS-PAGE and Western Blotting

Mouse hippocampal tissue samples were lysed in ice-cold RIPA buffer supplemented with phosphatase inhibitors [1 mM sodium orthovanadate, 1 mM phenylmethanesulfonyl fluoride (PMSF), 1 mM sodium pyrophosphate] and protease inhibitor Complete Mini Mixture (Roche, Indianapolis, IN, USA). Efficient lysis was achieved by pipetting up and down, passing through a 27-gauge needle and rotating at 4°C for at least 1 h. The lysed samples were centrifuged at 14,000 rcf for 15 min at 4°C. The supernatants were collected, the protein concentration in the supernatants was measured using Pierce BCA Protein Assay (Thermo Scientific, Waltham, MA, USA), and a mixture of Laemmli buffer (BioRad, Hercules, CA, USA) with β-mercaptoethanol (Sigma Aldrich) was added to the samples, following the BioRad's recommendations. The proteins (15–30 μg per well) were resolved by sodium dodecyl sulfate polyacrylamide gel electrophoresis (SDS-PAGE) on 10% tricine or glycine gels, and transferred to polyvinylidene difluoride (PVDF) membranes using traditional wet transfer or Trans-Blot Turbo Transfer System (BioRad, Hercules, CA, USA). The membranes were blocked by 2 h incubation in 5% bovine serum albumin (BSA) dissolved in Tris Buffered Saline supplemented with 0.1% Tween (TBST). Proteins were detected by immunoblotting with the following specific primary antibodies: mouse anti-β-actin [8H10D10] (3700S) and mouse anti-α-tubulin (3873S) from Cell Signaling Technology, Inc., rabbit anti-GFAP (ab7260), rabbit anti-P2RY12 [EPR18611] (ab184411), rabbit anti-CD68 (ab125212), and rabbit anti-APP C-terminus (Y188) (ab32136) from Abcam, Cambridge, MA, USA, and corresponding horseradish peroxidase (HRP)-conjugated secondary antibodies (7076S, 7074S from Cell Signaling Technology, Inc.). The membranes after immunoblotting were developed using Clarity ECL Western Blotting Substrate (BioRad, Hercules, CA, USA).

### Chemiluminescence Analysis and Densitometry

Immunoreaction signal was obtained through chemiluminescence in ChemiDoc™XRS + Imaging System with Image Lab™ Software (Bio-Rad). Semi-quantitative analysis of the band intensities, corresponding to the relative amounts of the proteins, was performed by densitometry using ImageJ v.1.46 software (Schneider et al., 2012).

### Statistical Analysis

Statistical analysis was performed in GraphPad Prism 7 software. The statistics from biochemical, morphological and Western blot analysis data are presented as an arithmetic mean ± standard deviation (SD) from at least three animals per experimental group (*n* = from 3 to 8). After examination of the normal distribution with the Shapiro-Wilk test, and the equality of variances, appropriate statistical tests have been selected. Statistical significance was determined using unpaired Student's *t*-test or one-way analysis of variance (ANOVA) followed by correction for multiple comparison with Bonferroni's post-test or unpaired, non-parametric Kruskal-Wallis test followed by Dunn's post-test with Bonferroni's correction for multiple comparisons, which are recommended in the situation when experimental groups differ in the total number of individuals per group and the number of individuals in groups is small. *P*-values from Student's *t*-test, one-way ANOVA and Kruskal-Wallis test are reported in Table 4, and from *post-hoc* multiple comparison tests in Supplementary Table 1. A *p*-value of < 0.05 was considered a predetermined threshold for statistical significance (^*^p < 0.05, ^**^p < 0.01, ^***^p < 0.001). All *p*-values for a specific statistical analysis are presented in Table 4 and Supplementary Table 1.

**Table 4:** Results of statistical analysis.

**Figure number: analyzed parameter**	**Age group**	**Statistical test**	**Significance**	***p*-Value**	**Value of** ** t/F/H statistic**
Figure 1A: Cholesterol	4M	One-way ANOVA	***	** <0.001**	*F*_(3, 16)_ = 22.5
	8M	One-way ANOVA	***	** <0.001**	*F*_(3, 17)_ = 36.2
	12M	One-way ANOVA	**	**0.005**	*F*_(3, 13)_ = 7.0
Figure 1B: Body weight	4M	One-way ANOVA	*	**0.015**	*F*_(3, 16)_ = 4.7
	8M	One-way ANOVA	***	** <0.001**	*F*_(3, 18)_ = 22.5
	12M	One-way ANOVA	ns	0.102	*F*_(3, 16)_ = 2.4
Figure 2D: Liver weight	4M	Unpaired *t*-Student	ns	0.088	*t*_(6)_= 2.0
	8M	Unpaired *t*-Student	**	**0.001**	*t*_(11)_= 4.4
	12M	Unpaired *t*-Student	ns	0.052	*t*_(9)_= 2.2
Figure 2E: Liver vs. body weight	4M	Unpaired *t*-Student	ns	0.761	*t*_(6)_= 0.3
	8M	Unpaired *t*-Student	**	**0.007**	*t*_(11)_= 3.3
	12M	Unpaired *t*-Student	ns	0.229	*t*_(9)_= 1.3
Figure 3A: GRA	4M	Kruskal-Wallis	ns	0.075	*H*_(3)_ = 6.6
	8M	One-way ANOVA	ns	0.233	*F*_(2, 14)_ = 1.6
	12M	One-way ANOVA	ns	0.853	*F*_(3, 15)_ = 0.3
Figure 3B:	4M	Kruskal-Wallis	ns	0.068	*H*_(3)_ = 6.7
MON	8M	Kruskal-Wallis	*	**0.024**	*H*_(2)_ = 6.7
	12M	Kruskal-Wallis	ns	0.918	*H*_(3)_ = 0.6
Figure 3C: LYM	4M	One-way ANOVA	ns	0.374	*F*_(3, 16)_ = 1.1
	8M	One-way ANOVA	ns	0.073	*F*_(2, 14)_ = 3.2
	12M	One-way ANOVA	ns	0.659	*F*_(3, 15)_ = 0.7
Figure 3D:	4M	One-way ANOVA	ns	0.136	*F*_(3, 16)_ = 2.1
WBC	8M	Kruskal-Wallis	ns	0.288	*H*_(3)_ = 3.8
	12M	One-way ANOVA	ns	0.695	*F*_(3, 15)_ = 0.5
Figure 4A:	4M	One-way ANOVA	***	** <0.001**	*F*_(3, 16)_ = 13.6
GFAP	8M	One-way ANOVA	ns	0.130	*F*_(3, 17)_ = 2.2
	12M	Kruskal-Wallis	ns	0.982	*H*_(3)_ = 0.8
Figure 5A:	4M	Kruskal-Wallis	***	** <0.001**	*H*_(3)_ = 19.2
Iba1	8M	Kruskal-Wallis	***	** <0.001**	*H*_(3)_ = 37.7
	12M	Kruskal-Wallis	***	** <0.001**	*H*_(3)_ = 19.1
Figure 5B: P2RY12	4M	One-way ANOVA	ns	0.529	*F*_(3, 16)_ = 0.8
	8M	Kruskal-Wallis	*	**0.045**	*H*_(3)_ = 8.0
	12M	Kruskal-Wallis	*	**0.023**	*H*_(3)_ = 9.5
Figure 5C: CD68	4M	One-way ANOVA	ns	0.391	*F*_(3, 16)_ = 0.4
	8M	Kruskal-Wallis	ns	0.172	*H*_(3)_ = 5.0
	12M	Kruskal-Wallis	**	**0.003**	*H*_(3)_ = 13.8
Figure 6B: APP full-length	4M	One-way ANOVA	**	**0.005**	*F*_(3, 16)_ = 6.4
	8M	One-way ANOVA	ns	0.085	*F*_(3, 18)_ = 2.6
	12M	One-way ANOVA	ns	0.051	*F*_(3, 15)_ = 3.3
Figure 6B: APP CTFs	4M	One-way ANOVA	**	**0.001**	*F*_(3, 16)_ = 8.5
	8M	One-way ANOVA	ns	0.287	*F*_(3, 18)_ = 1.4
	12M	One-way ANOVA	ns	0.161	*F*_(3, 15)_ = 2.0

*ns, non-significant*;

* *p < 0.05*;

** *p < 0.01*;

*** *p < 0.001. Bold values are intended to represent statistically significant values*.

## Results

### Final Experimental Groups

As described in Materials and Methods, in the study four experimental mouse groups were used: (1) CTR, (2) LPS, (3) WD, and (4) WD + LPS. Each group of animals was analyzed at five ages: 4M, 8M, 12M, 16M, and 20M. As verified in the following chapters, control APPswe mice from 4M to 12M correspond to the early, pre-plaque stages of AD, before amyloid plaque formation, which started not earlier than in 16M. Precise information about applied statistical tests and obtained values from particular analysis are reported in Table 4 and Supplementary Table 1. Key values from one-way ANOVA and Kruskal-Wallis tests have been also reported in the main text, and from multiple comparison *post-hoc* tests on the figures.

### Western Diet Induces Metabolic Disorders, Such as Hypercholesterolemia, Obesity, Non-alcoholic Fatty Liver Disease, and Non-alcoholic Steatohepatitis

**Figure 1:**
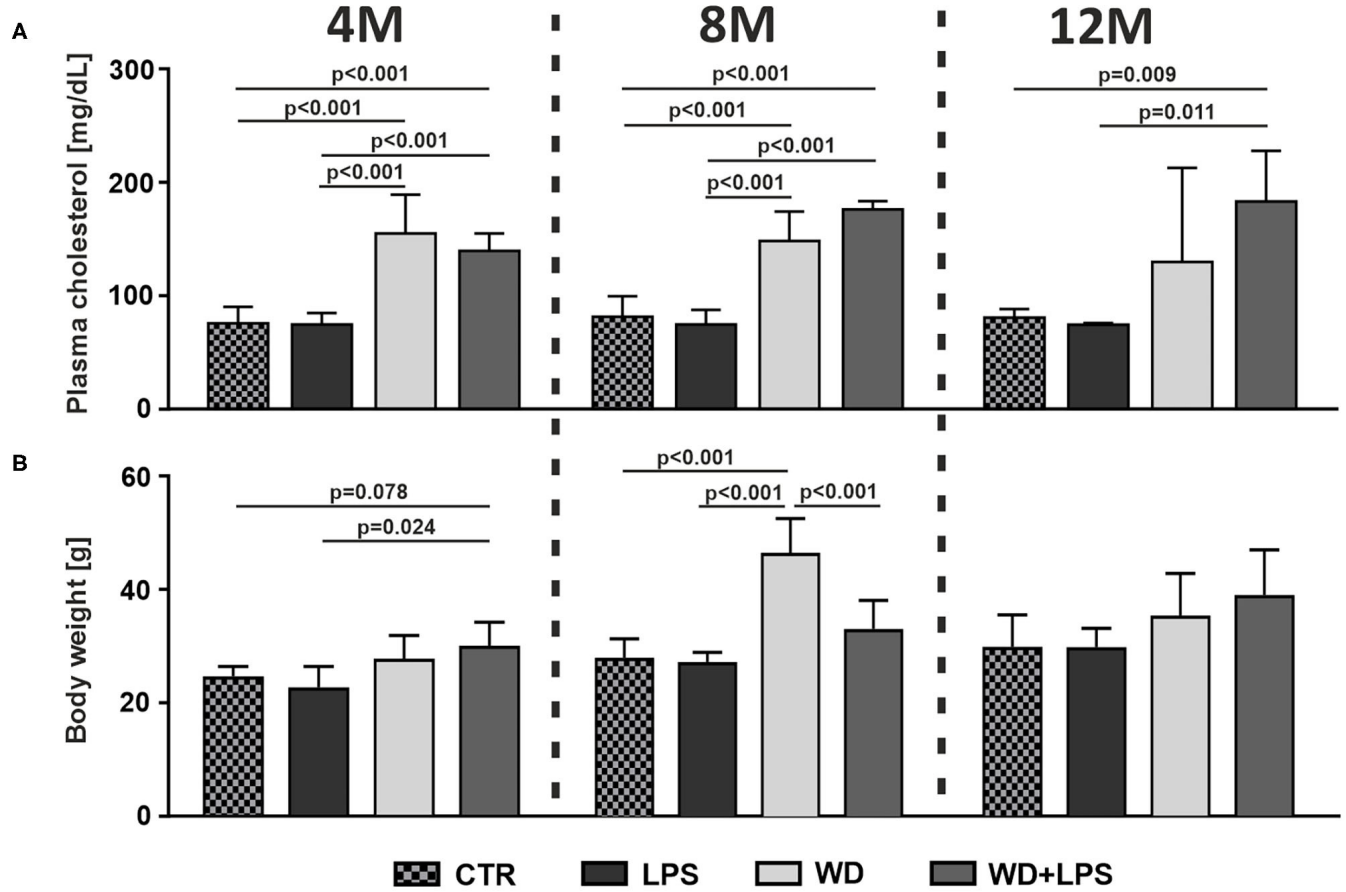
Western diet induces hypercholesterolemia (HChol) and obesity. Graphs show Western diet-induced increases of **(A)** plasma cholesterol level, and **(B)** body weight, in 4-, 8-, and 12-month-old APPswe mice in WD and WD + LPS groups compared to CTR and LPS. All the data are presented as arithmetic mean ± SD (*n* = from 3 to 8 animals per experimental group). The statistics were calculated using unpaired ANOVA with Bonferroni's *post-hoc* test. A *p*-value of <0.05 was considered a predetermined threshold for statistical significance.

First we analyzed how WD affects metabolic parameters in APPswe mice. As shown in Figure 1A, short-term WD feeding (3 weeks) resulted in induction of HChol already in young, 4M animals. Comparison of WD or WD + LPS groups to CTR and LPS groups clearly demonstrated a very significant increase of plasma cholesterol levels as early as in 4M animals on WD (Figure 1A: 4M [*F*_(3, 16)_ = 22.5, *p* < 0.001]). This HChol was maintained in 8M and 12M mice fed with WD, i.e., after 5 and 9 months of WD feeding (Figure 1A: 8M [*F*_(3, 17)_ = 36.2, *p* < 0.001], 12M [*F*_(3, 13)_ = 7.0, *p* = 0.005]). Similarly, 3 weeks of WD feeding increased the body weight in young, 4M mice in WD and in WD + LPS compared to CTR and LPS groups (Figure 1B: 4M [*F*_(3, 16)_ = 4.7, *p* = 0.015]). Observed body weight gain was deepened during further continuous WD feeding, resulting in obesity in 8M mice (Figure 1B: 8M [*F*_(3, 18)_ = 22.5, *p* < 0.001]). In the 12M groups, the WD-fed mice still showed some higher body weight compared to controls; smaller differences in the mean body weight between all groups at 12M appear to be caused by visible aging-related increases in the body weight in CTR and LPS groups (Figure 1B: 12M [*F*_(3, 16)_ = 2.4, *p* = 0.102]).

**Figure 2:**
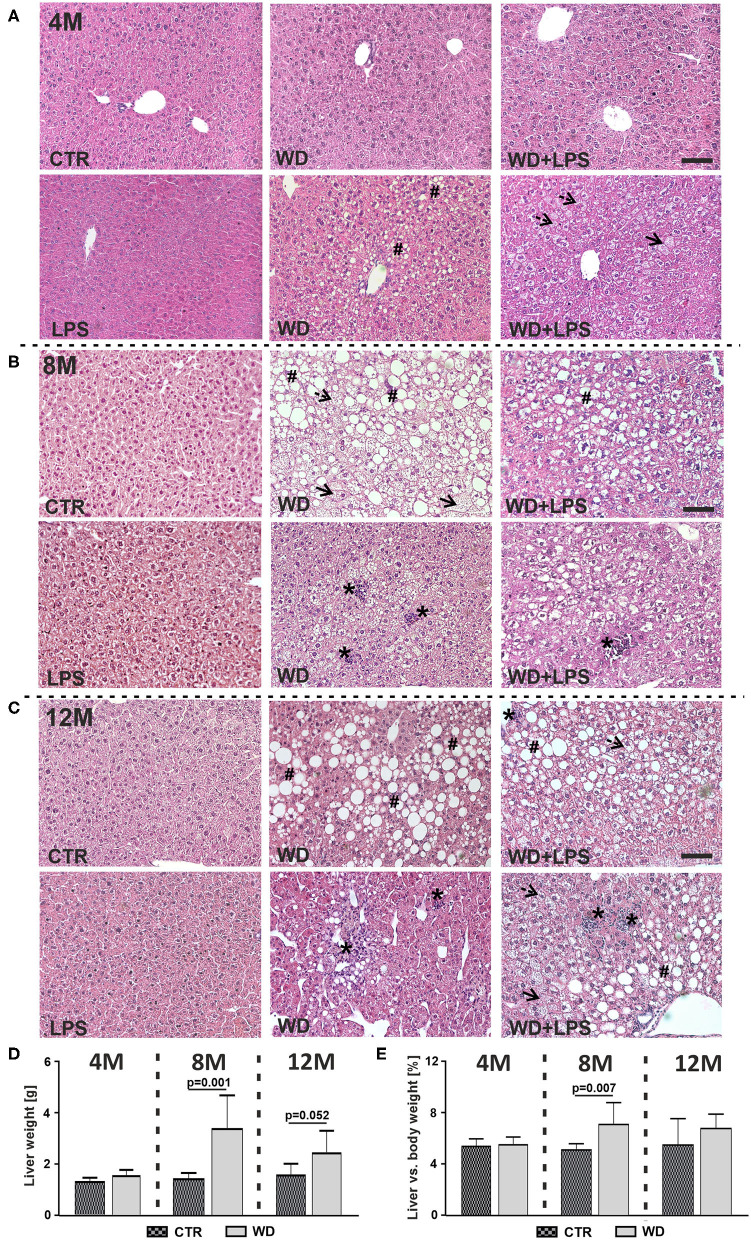
Western diet induces non-alcoholic fatty liver disease (NAFLD) and non-alcoholic steatohepatitis (NASH). Microscopic images of hematoxylin-eosin histological staining of liver tissue from **(A)** 4-month-old APPswe mice fed with WD for 3 weeks, **(B)** 8-month-old APPswe mice fed with WD for 5 months, **(C)** 12-month-old APPswe mice fed with WD for 9 months; dotted arrows—microvesicular steatosis (discrete round lipid vacuoles within hepatocytes), arrows—hypertrophic hepatocytes, #—hepatocellular ballooning and macrovesicular steatosis, *–lobular inflammation; scale bars correspond to 50 μm; magnification: ×20. **(D,E)** Graphs show comparison of **(D)** liver weight in WD and CTR mice at 4-, 8-, and 12-months of age and **(E)** the liver vs. body weight ratio in WD and CTR mice 4-, 8-, and 12-months of age. Data are presented as arithmetic mean ± SD (*n* = from 3 to 8 animals per experimental group). The statistics were calculated using unpaired Student *t*-test. A *p*-value of <0.05 was considered a predetermined threshold for statistical significance.

NAFLD is characterized by hepatic fat accumulation and infiltration of immune cells in the liver parenchyma, leading to cytokine release causing hepatocytes injury and subsequent liver pathology characteristic for NASH both in humans (Friedman et al., 2018; Morsiani et al., 2019) and mice (Tojima et al., 2012; Liang et al., 2014). In our study, WD feeding resulted in a marked lipid droplet deposition and microvesicular steatosis observed as discrete round lipid vacuoles within hepatocytes (Figure 2, dotted arrows), hypertrophic hepatocytes (Figure 2, arrows), hepatocellular ballooning and macrovesicular steatosis (Figure 2, #). Moreover, WD induced lobular inflammation revealed as deposits of inflammatory cells (Figure 2, ^*^), indicating NASH. These signs of liver damage were observed gradually, starting as early as in 4M WD as well as WD + LPS mice and were not found in CTR and LPS animals. Three weeks of WD feeding induced mainly microvesicular steatosis and mild macrovesicular steatosis characterized by small size (Figure 2A). Significant liver damage was observed after 5 months of WD feeding, i.e., at 8M hepatocellular ballooning with macrovesicular steatosis and strong inflammatory cells infiltration in the liver parenchyma (Figure 2B). These morphological changes dramatically deepened after 9 months of WD feeding, in the 12M mice (Figure 2C).

Despite the severe hepatocytes damage, a large increase in liver weight was found in 8M mice fed with WD (Figure 2D: 8M [*t*_(11)_ = 4.4, *p* = 0.001]). Graphs in Figures 2D,E show that observed liver weight gain constitutes a significant proportion of body weight gain with significant increase in 8M mice fed with WD (Figure 2E: 8M [*t*_(11)_ = 3.3, *p* = 0.007]). Altogether, these data demonstrate WD-induced development of extensive fatty liver with profound hepatocytes degradation, characteristic of the late stages of severe NAFLD and NASH (Lindenmeyer and McCullough, 2018). The differences in liver weight/body weight ratios between WD and CTR groups were not as pronounced in 12M mice (Figure 2D: 12M [*t*_(9)_ = 2.2, *p* = 0.052], Figure 2E: 12M [*t*_(9)_ = 1.3, *p* = 0.229]). This seems due to body and liver weight gain related to aging in the control group.

### Western Diet Is Associated With Signs of Low-Grade Systemic Inflammation

**Figure 3:**
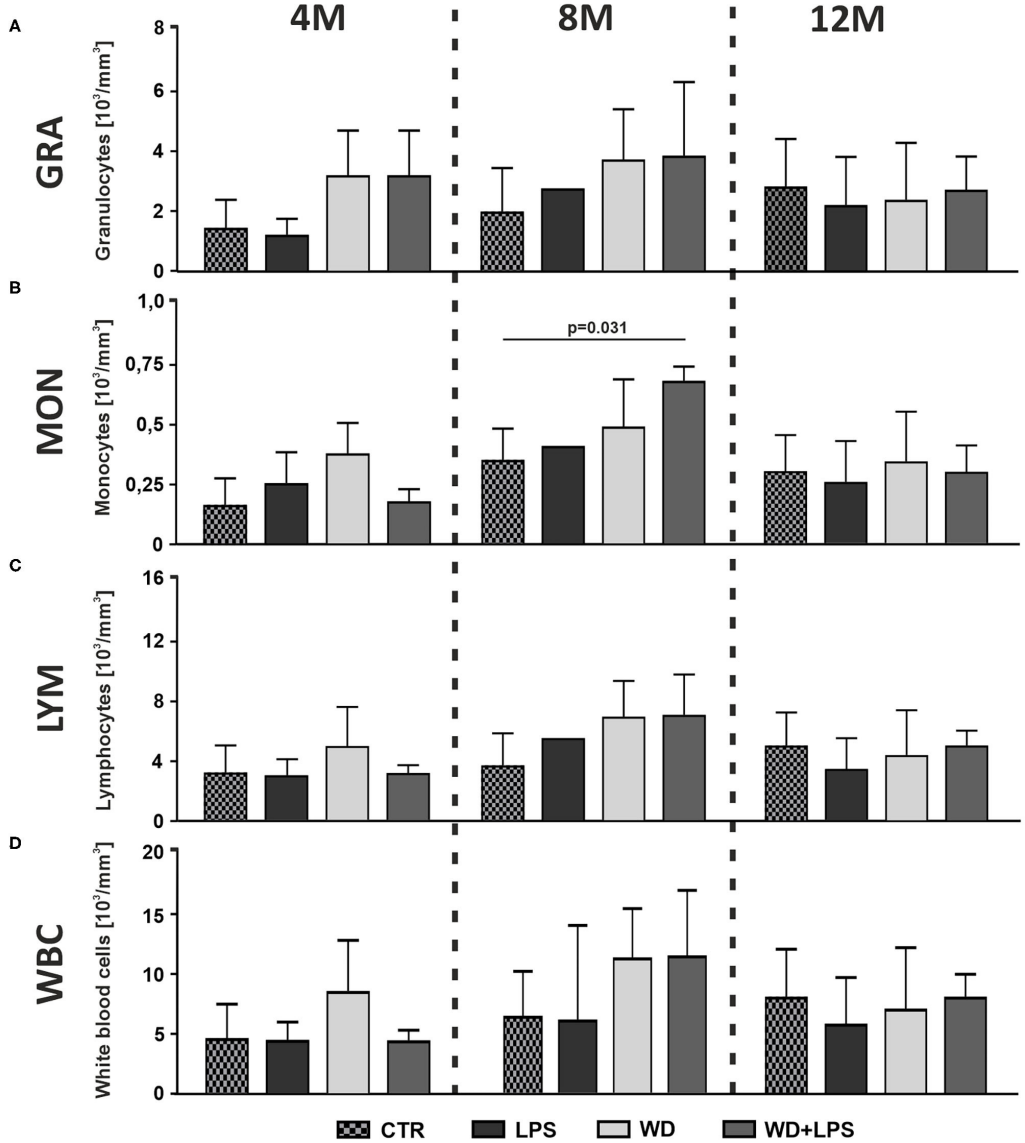
Western diet induces low-grade systemic inflammation. Graphs show WD-induced increases of **(A)** blood granulocytes (GRA) concentration in 4- and 8-month-old APPswe mice, **(B)** monocytes (MON) concentration in 4- and 8-month-old APPswe mice, **(C)** lymphocytes (LYM) concentration in 8-month-old APPswe mice and **(D)** total white blood cells (WBC) concentration in 8-month-old APPswe mice. All the data are presented as arithmetic mean ± SD (*n* = from 3 to 8 animals per experimental group with exception in 8M LPS groups where means were calculated based on *n* = 2, and these values were not included in statistical analysis). The statistics were calculated using one-way analysis of variance (ANOVA) followed by Bonferroni's post-test or non-parametric Kruskal-Wallis test followed by Dunn's post-test. A *p*-value of <0.05 was considered a predetermined threshold for statistical significance.

In order to assess whether diet-induced inflammation in the metabolic organ tissues coexists with signs of inflammation in circulating blood, we performed hematological and morphological analysis of blood. The innate immune system is the first line of defense against initial environmental challenges, and is activated much more rapidly than the adaptive immune system. Granulocytes (GRA) comprise 60–70% of blood leucocytes, and more than 90% of granulocytes are neutrophils, making up the largest fraction of white blood cells (WBC). Neutrophils are typically the first immune cells to respond to inflammation and can exacerbate the chronic inflammatory state by helping to recruit macrophages and by interacting with antigen-presenting cells (Talukdar et al., 2012). In line with this, we observed that 3 weeks of WD already caused some non-significant increase of blood GRA concentration in 4M mice in WD and WD + LPS groups compared to control CTR and LPS groups (Figure 3A: 4M [*H*_(3)_ = 6.6, *p* = 0.075]). The same increasing trend in GRA levels continued after 5 months of WD feeding in both WD and WD + LPS groups (Figure 3A: 8M [*F*_(2, 14)_ = 1.6, *p* = 0.233]). Similarly, 3 weeks of WD induced a non-significant increase of blood monocytes concentration (MON) (Figure 3B: 4M [*H*_(3)_ = 6.7, *p* = 0.068]). The increase was maintained in 8M mice from WD and WD + LPS (Figure 3B: 8M [*H*_(2)_ = 6.7, *p* = 0.024]). Also, a tendency to an increase of blood lymphocytes concentration (LYM) was observed (Figure 3C: 8M [*F*_(2, 14)_ = 3.2, *p* = 0.073]), which suggests that WD induced some activation of the adaptive immune system. An increase of total WBC concentration in the blood, comprising various white cell subpopulations, appeared in 4M and 8M mice, in the WD and WD + LPS groups (Figure 3D: 4M [*F*_(3, 16)_ = 2.1, *p* = 0.136], 8M [*H*_(3)_ = 3.8, *p* = 0.288]), but the changes were not significant. In turn, WD in 12M mice led to some non-significant reduction in the concentration of GRA, MON, LYM and WBC in the blood (Figure 3A: 12M [*F*_(3, 15)_ = 0.3, *p* = 0.853], Figure 3B: [*H*_(3)_ = 0.6, *p* = 0.918], Figure 3C: [*F*_(3, 15)_ = 0.7, *p* = 0.659], Figure 3D: [*F*_(3, 15)_ = 0.5, *p* = 0.695]). In the light of WD-induced metabolic disturbances (Figure 2), this reduction may have been triggered by infiltration of immune cells into adipose tissue, liver, and brain, where immune cells secrete a number of pro-inflammatory cytokines and chemokines to counteract metabolic damage (Xu et al., 2014; Shukla et al., 2019; Liu et al., 2020). In contrast to the leucocyte parameters (Figure 3), the global analysis of such basic erythrocyte parameters as red blood cells count, anisocytosis indicator and mean cell volume did not show any changes between tested groups (data not shown). Overall, the hematological analysis of blood demonstrated some non-significant increases in the levels of WBC, especially GRA and MON, after only 3 weeks of WD feeding, and in LYM after 5 months of WD. The trends in increases observed in blood cell subpopulations cannot be ignored despite the lack of significance but should be evaluated in the context of inflammatory processes detected in the metabolic tissues, such as liver because white blood cells infiltrate the inflamed liver tissue. Moreover, given that there are no clear criteria for defining low-grade systemic inflammation, its detection cannot be based only on significant differences in blood morphology parameters, but should also consider shifts of the analyzed parameters to the upper limits of the norms, as shown in our study. Taking this into account, WD-induced trends in increased leucocyte levels in the context of concurrence with HChol, obesity, and NAFLD support the detection of low-grade systemic inflammation.

### Western Diet Induces Neuroinflammation and Contributes to the Impairment in Astroglia and Microglia Activation

**Figure 4:**
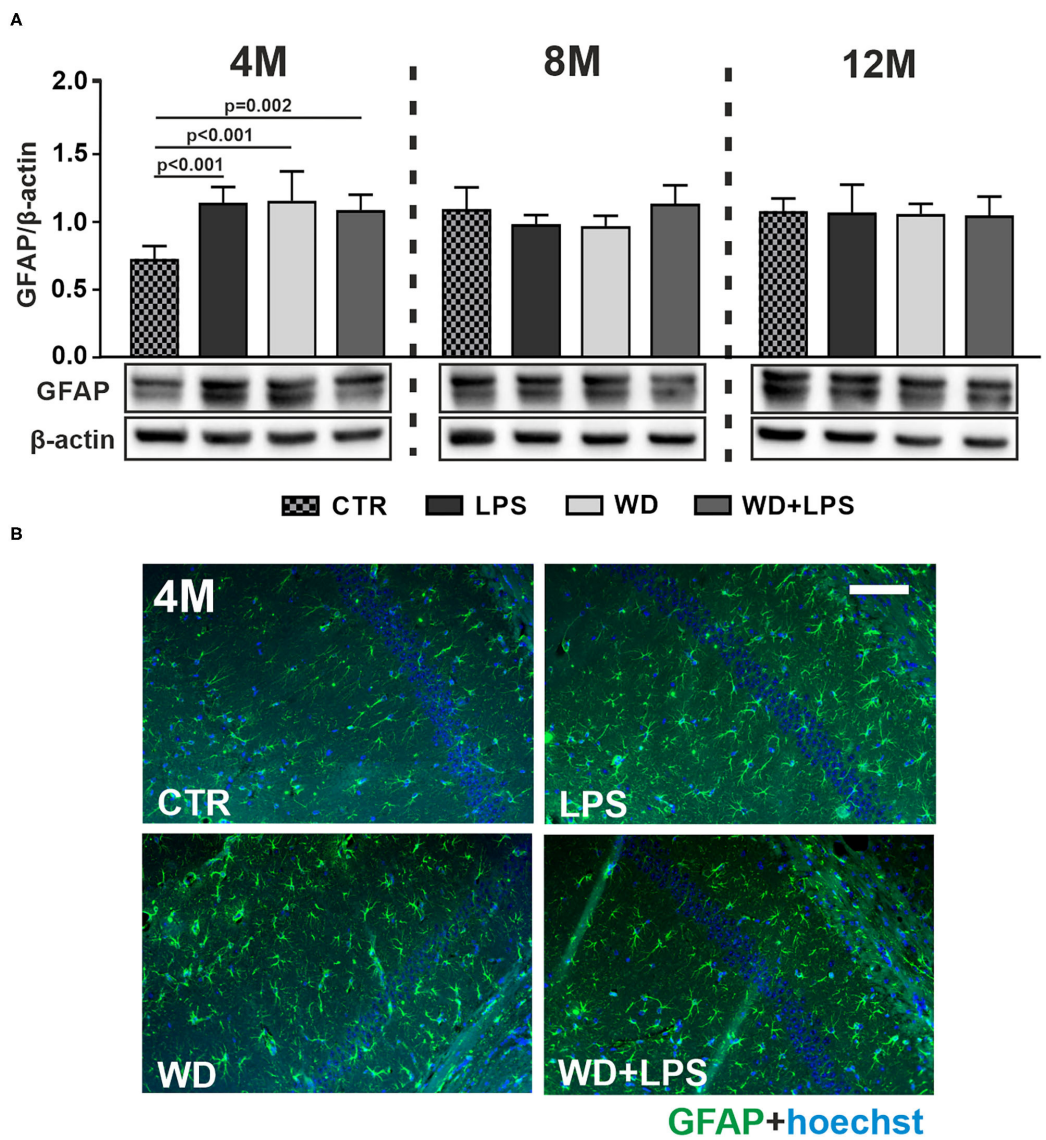
Western diet accelerates astrogliosis in APPswe mouse hippocampus. **(A)** Graphs show mean GFAP levels detected by immunoblotting in the hippocampal tissue lysates from CTR, LPS, WD, and WD + LPS groups of mice at 4-, 8-, and 12-months of age. Below are representative immunoblots. WD caused increased GFAP levels in the hippocampus of 4-month-old APPswe mice. Data are presented as arithmetic mean of GFAP/actin ± SD (*n* = from 4 to 8 animals per experimental group). The statistics were calculated using one-way analysis of variance (ANOVA) followed by Bonferroni's post-test or non-parametric Kruskal-Wallis test followed by Dunn's post-test. A *p*-value of <0.05 was considered a predetermined threshold for statistical significance. **(B)** Representing immunofluorescence images show the increases in GFAP staining in the hippocampus in WD and WD + LPS mice compared to CTR mice. All mice were 4-month-old; the scale bar corresponds to 50 μm; magnification ×20; green fluorescence—GFAP, blue fluorescence—Hoechst (nuclei).

Three weeks of WD feeding enhanced astrogliosis in the hippocampal brain region as revealed by increased levels of the astrocyte marker glial fibrillary acidic protein (GFAP) detected by immunoblotting in the young, 4M APPswe mice in WD and WD + LPS groups compared to CTR group (Figure 4A: 4M [*F*_(3, 16)_ = 13.6, *p* < 0.001]). Similarly enhanced GFAP levels were observed in LPS mice compared to CTR mice (Figure 4A: 4M [*F*_(3, 16)_ = 13.6, *p* < 0.001]). Thus, short-term WD feeding caused astrogliosis at a comparable level to that induced by LPS injection, suggesting that WD acts as an agent comparable to LPS, a well-characterized, infection-derived, acute-neuroinflammatory agent. Moreover, Figure 4 demonstrates that WD, like LPS, accelerated astrogliosis in the hippocampus of this AD mouse model: the level of astrogliosis observed after a short period of WD feeding in 4M animals was comparable to the level induced by mutation in APP in older, i.e., 8M and 12M APP control mice. These data indicate that WD accelerated astrocyte activation by 4 months. Furthermore, immunoblotting data in Figure 4 showed no differences among all groups in GFAP levels in the hippocampal tissue of 8M and 12M mice. This suggests that the activated astroglia reached a constant level and underwent an adaptive state (Figure 4A: 8M [*F*_(3, 17)_ = 2.2, *p* = 0.130], 12M [*H*_(3)_ = 0.8, *p* = 0.982]). All the described changes were also reflected in the qualitative analysis of hippocampal tissue using immunofluorescence (Figure 4B and Supplementary Figure 1).

**Figure 5:**
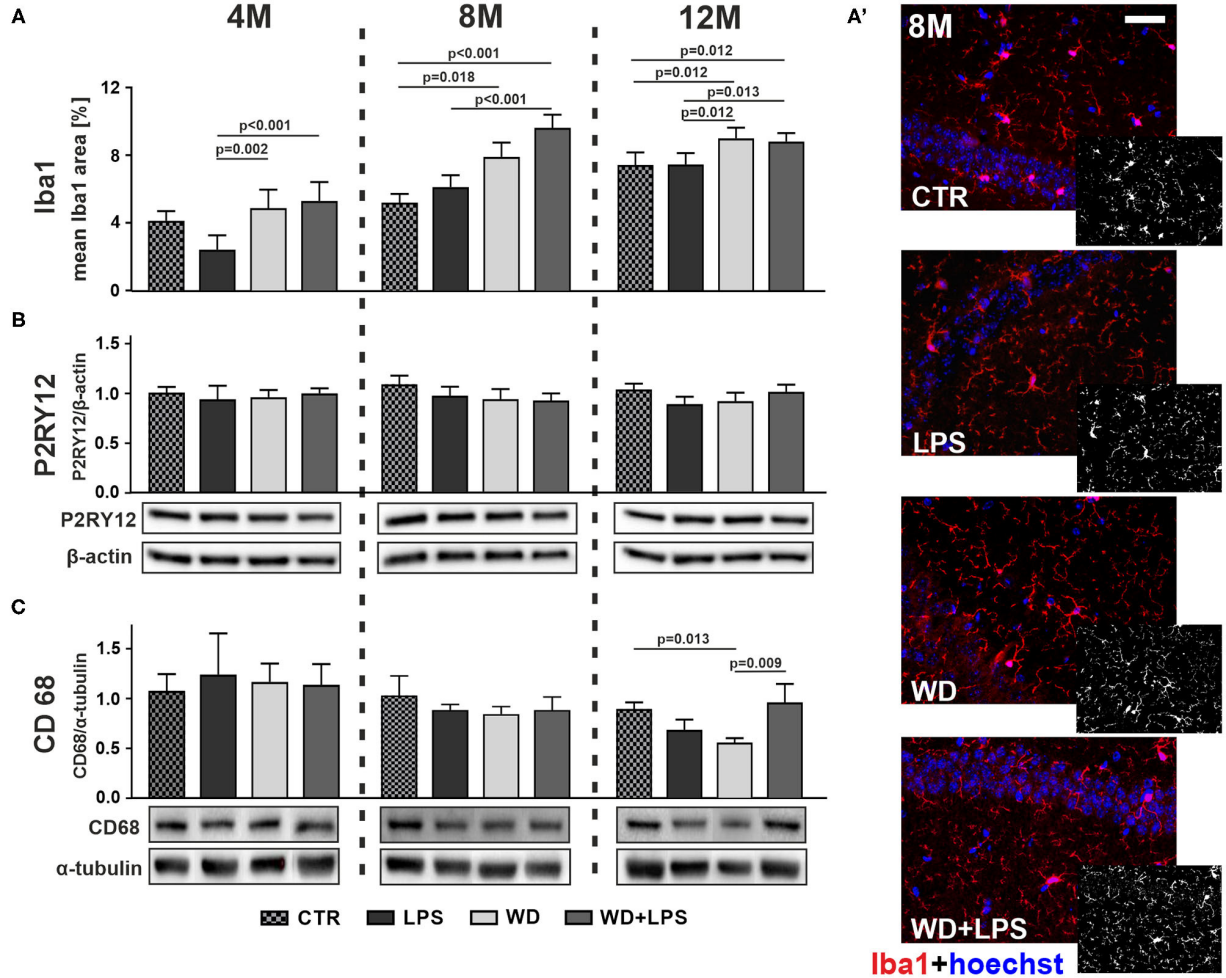
Western diet accelerates hippocampal microglia activation and impairs their phagocytic function. **(A)** Graphs show mean areas of the microglia activation marker Iba1 positive staining detected in hippocampal tissue by immunofluorescence microscopy in CTR, LPS, WD, and WD + LPS groups of APPswe mice at 4-, 8-, and 12- months of age. All the data are presented as the arithmetic mean ± SD of Iba1 positive staining area from many different parts and cross-section of the hippocampi from 1 or 2 representative animals per group. The statistics were calculated using non-parametric Kruskal-Wallis test followed by Dunn's post-test. A *p*-value of <0.05 was considered a predetermined threshold for statistical significance. Graphs demonstrate that Iba1 immunostaining increased in WD and WD + LPS groups compared to both control groups and that the most significant increase was detected in 8-month-old mice. **(A')** Representative immunofluorescence images of the data shown in A present the increases in Iba1 positive staining in hippocampal tissue from 8-month-old APPswe mice in WD and WD + LPS groups compared to CTR and LPS groups; the scale bar corresponds to 50 μm; magnification: ×20; red fluorescence—Iba1, blue fluorescence—Hoechst (nuclei). Graphs show mean protein levels detected by immunoblotting of microglial anti-inflammatory homeostatic marker P2RY12 **(B)** and microglial phagocytic marker CD68 **(C)** in the mouse hippocampal tissue lysates from CTR, LPS, WD, and WD + LPS groups at the age of 4, 8, and 12 months. Below are representative immunoblots. Immunoblotting results show no differences in P2RY12 hippocampal levels among all experimental groups in 4-, 8-, and 12-month-old APPswe mice **(B)**, and decrease of hippocampal CD68 level in 12-month-old WD mice **(C)**. All the data from P2RY12 and CD68 markers are presented as arithmetic mean ± SD (*n* = from 4 to 8 animals per experiment). Statistical data in **(B,C)** were calculated using one-way analysis of variance (ANOVA) followed by Bonferroni's post-test or non-parametric Kruskal-Wallis test followed by Dunn's post-test. A *p*-value of <0.05 was considered a predetermined threshold for statistical significance.

Immunofluorescence analysis in Figure 5A showed that WD after 5 and 9 months of feeding resulted in significantly enhanced microglial activation in the hippocampus, as indicated by increased immunofluorescence staining area of ionized calcium-binding adapter molecule 1 (Iba1), which is a microglial activation marker, in 8M and 12M mice from WD and WD + LPS groups compared to both control groups: standard diet (CTR) group and LPS treated group (Figure 5A: 8M [*H*_(3)_ = 37.7, *p* < 0.001], 12M [*H*_(3)_ = 19.1, *p* < 0.001], Figure 5A' and Supplementary Figure 2). Of note, compared to young 4M animals, in 12M mice Iba1 positive area increased in hippocampal tissue from CTR and LPS groups demonstrating aging-related microglial activation. Nevertheless, even in the 12M animals, the hippocampal Iba1 positive areas in mice from WD and WD + LPS groups were still significantly larger than in age-matched mice from CTR and LPS groups (Figure 5A: 12M [*H*_(3)_ = 19.1, *p* < 0.001]). There were no significant changes in Iba1 hippocampal levels after 3 weeks of WD feeding compared to CTR mice fed with standard diet in contrast to LPS treatment (Figure 5A: 4M [*H*_(3)_ = 19.2, *p* < 0.001]). Altogether, the results in Figure 5 demonstrate that compared to standard diet, WD feeding enhanced and accelerated microglial activation by 4 months, causing the largest increase in Iba1 levels after 5 months of WD, and persisting during aging in the older animal groups (Figure 5A: 8M, 12M). Figure 5 shows also that compared to the CTR group, intraperitoneal injection of LPS did not significantly increase Iba1 levels in all age groups, and even caused some lowering of Iba1 levels in 4M animals (Figure 5A: 4M [*H*_(3)_ = 19.2, *p* < 0.001]).

While the Iba1 marker is widely used to detect microglial activation, it is not sufficient to indicate microglial polarization state because Iba1 is expressed both in pro-inflammatory and anti-inflammatory microglia (M1 and M2, respectively). Generally, it is difficult to find a marker unique for pro-inflammatory resident microglia because of a very high similarity of pro-inflammatory markers on microglia and on activated monocyte-derived macrophages (MDM) which infiltrate the brain from the periphery during pathological conditions (Walker and Lue, 2015; Orihuela et al., 2016). Instead we decided to look at an anti-inflammatory marker: P2RY12, known to be highly-specific for resident, homeostatic, microglia. P2RY12 has been defined as a specific marker to discriminate between microglia, with high levels of expression, and macrophages (Butovsky et al., 2014; Mildner et al., 2017). In our research WD did not change the level of P2RY12 in the hippocampus of 4M, 8M, and 12M APP mice (Figure 5B: 4M [*F*_(3, 16)_ = 0.8, *p* = 0.529], 8M [*H*_(3)_ = 8.0, *p* = 0.045 with no significant differences in *post-hoc* multiple comparison test], 12M [*H*_(3)_ = 9.5, *p* = 0.023 with no significant differences in *post-hoc* multiple comparison test]). This suggests that WD-induced activation of microglia is not associated with anti-inflammatory polarization, but rather with a pro-inflammatory profile.

Further we investigated whether WD-induced activation of microglia affected basic microglial phagocytic function. To this aim we analyzed the level of CD68, an indicator of microglial phagocytic activity present in both M1 and M2 microglia (Martinez et al., 2013; Walker and Lue, 2015). The phagocytic function of microglia is crucial to clear accumulated Aβ debris and plaques in humans (Zotova et al., 2011, 2013). As demonstrated in Figure 5C, Long-term WD feeding led to decreased hippocampal CD68 levels in 12M animals compared to controls fed standard diet (Figure 5C: 12M [*H*_(3)_ = 13.8, *p* = 0.003]). These data suggest that in the brains of WD-fed APPswe mice activated pro-inflammatory microglia cells were losing their abilities to efficiently phagocytose Aβ debris and deposits. This inefficient removal of Aβ deposits probably accelerated amyloid plaque formation, so we decided to verify this hypothesis by measuring immunofluorescence of Aβ.

### Western Diet Accelerates Aβ Accumulation and Plaques Formation in the Hippocampus and Increases Levels of Amyloid Precursor Protein

**Figure 6:**
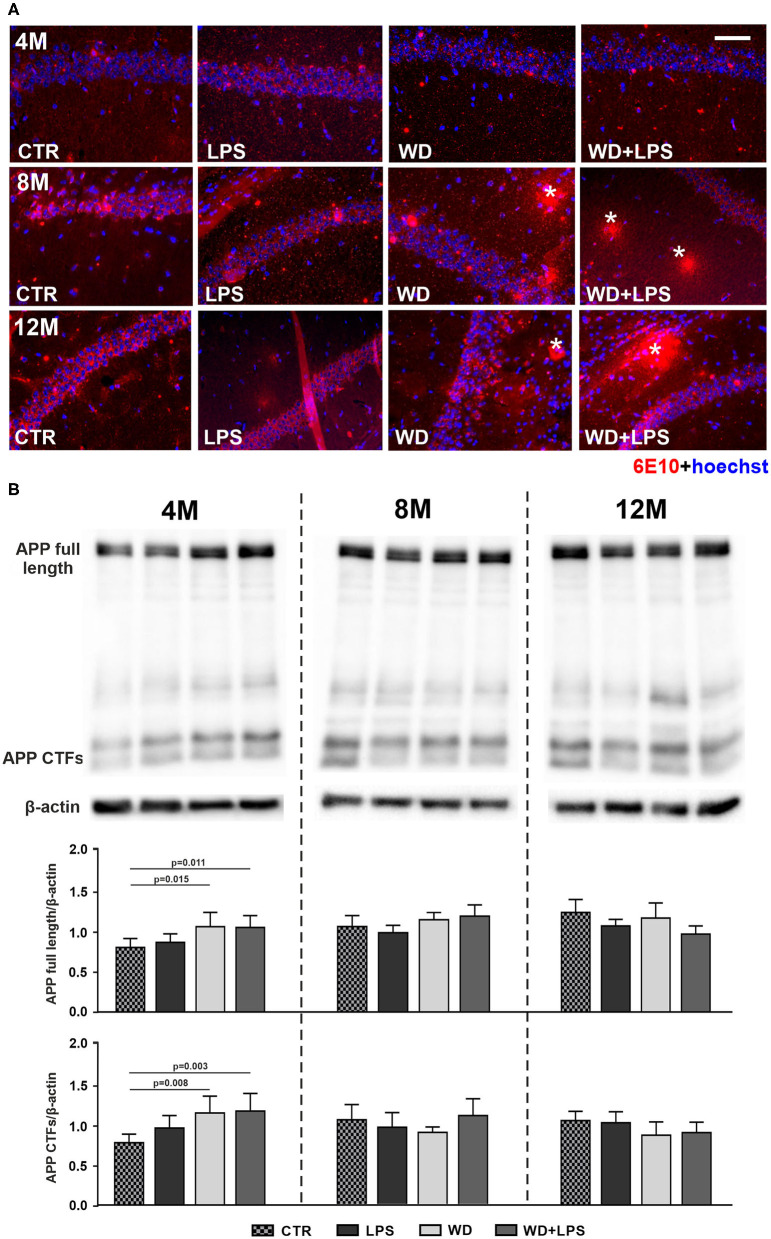
Western diet accelerates deposition of amyloid-β plaques and APP accumulation in the hippocampus. **(A)** Comparative immunofluorescence images of brain hippocampal tissue from 4-, 8-, and 12-month-old APPswe mice probed with anti-Aβ antibody 6E10 show accelerated formation of Aβ plaques (*) in the hippocampus of 8-month-old APPswe mice fed with WD (WD and WD + LPS groups compared to CTR and LPS control groups). In control APPswe mice fed with standard diet (CTR) or treated with LPS, Aβ plaques were not observed even at the age of 12 months, and as shown in Figure 3 were detected as late as in 16-month-old mice group; the scale bar corresponds to 50 μm; lens magnification ×20; red fluorescence—6E10 (amyloid-β staining), blue fluorescence—Hoechst (nuclei). **(B)** Graphs show increased levels of APP full-length and its C-terminal end fragments in WD and WD + LPS experimental groups in 4-month-old APPswe mouse hippocampus obtained in Western blot analysis. All the data are presented as arithmetic mean of APP full-length/actin and CTFs/actin ± SD (*n* = from 4 to 8 animals per experimental group). The statistics were calculated using one-way analysis of variance (ANOVA) followed by Bonferroni's post-test. A *p*-value of <0.05 was considered a predetermined threshold for statistical significance.

**Figure 7:**
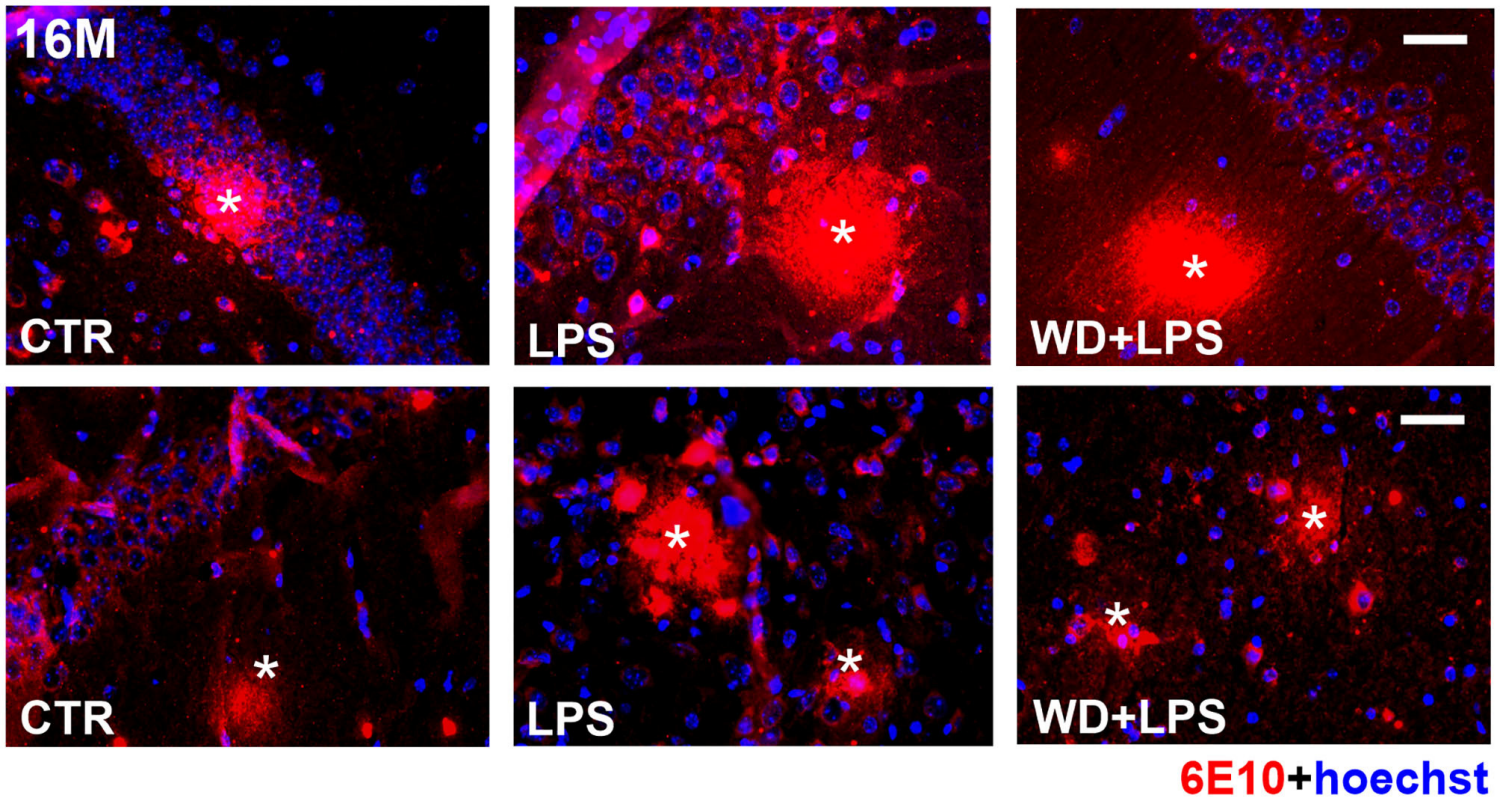
In 16-month-old APPswe mice deposition of amyloid-β plaques in the hippocampus occurs in all experimental groups including animals fed standard diet. The microphotographs show immunofluorescence labeling of Aβ (6E10) in the brain hippocampal tissue of 16-month-old APPswe mice from CTR, LPS, and WD + LPS experimental groups. Aβ plaques (*) are present in all experimental groups at this age; the scale bar corresponds to 50 μm; lens magnification ×20; red fluorescence—6E10 (amyloid-β), blue fluorescence—Hoechst (nuclei).

Our immunofluorescence analysis of the mouse hippocampus showed Aβ plaque formation after as little as 5 months of WD feeding (Figure 6A). Aβ plaques were detected in the hippocampus of WD and WD + LPS mice first at the age of 8M and 12M (Figure 6A: 8M, 12M). In control CTR and LPS mice Aβ plaques were not detected until the animals reached the age of 16 months, i.e., 8 months later (Figure 7). Due to particularly high mortality among old animals fed with WD, no animals in the WD group reached 16-months of age, but some mice in the WD + LPS group survived, presenting accumulation of Aβ plaques in the hippocampus (Figure 7). Compared to well-characterized control APPswe mice in which amyloid plaques started to be detected at 16-months of age, WD accelerated Aβ accumulation and plaque formation in the hippocampus meaning that in mice fed WD amyloid plaques started to be detected not at 16- but at 8-months of age. Aβ peptides are generated from amyloid precursor protein (APP) in the amyloidogenic pathway and deposited as plaques (Jäger et al., 2009; Hare, 2010; Haass et al., 2012). In healthy individuals, most of the APP is constitutively cleaved by α-secretase generating the soluble ectodomain of APP (sAPPα) and a truncated C-terminal peptide (CTF-α or C-83). In the amyloidogenic pathway, APP is proteolyzed by β-secretase (BACE-1) forming the membrane bound C-terminal fragment (CTF-β or C-99), which is further cleaved by the γ-secretase leading to Aβ production and plaque formation. Our Western blotting analysis of APP and its CTFs showed that 3 weeks of WD feeding increased the hippocampal APP level (Figure 6B: APP full 4M [*F*_(3, 16)_ = 6.4, *p* = 0.005]), and its CTFs (Figure 6B: CTFs 4M [*F*_(3, 16)_ = 8.5, *p* = 0.001]), demonstrating enhanced cleavage of APP. These processes were observed 4 months earlier in WD fed mice than in mice fed standard diet (CTR or LPS mice) (Figure 6B: 8M). In all 8M and 12M experimental groups the levels of APP and its CTFs were similar (Figure 6B: APP full 8M [*F*_(3, 18)_ = 2.6, *p* = 0.085] and 12M [*F*_(3, 15)_ = 3.3, *p* = 0.051], CTFs 8M [*F*_(3, 18)_ = 1.4, *p* = 0.287], and 12M [*F*_(3, 15)_ = 2.0, *p* = 0.161]). These data suggest that WD feeding impacts *APP* gene transcription and influences the activity of secretases, and dysregulates the glial cell activity and accumulated Aβ clearance resulting in accelerated amyloid plaque formation.

Some additional support for development of amyloidopathy in the established “metabolic” mouse model of AD was provided by comparison of amyloidopathy in the brain hippocampal tissue in 20M WD + LPS APPswe mice to the post-mortem brain tissue of in the 85-year-old human patient diagnosed with SAD (Supplementary Figure 3). We observed that long-term WD feeding led to the development of neuropathological changes associated with highly advanced amyloidopathy indistinguishable from those observed in the brain of SAD patient. Overall, the data collected in WD-treated APPswe mice suggest that the mouse model may be a useful tool for future research on participation of metabolic factors and peripheral processes in development and progression of AD.

### Summary of the Obtained Results

**Figure 8:**
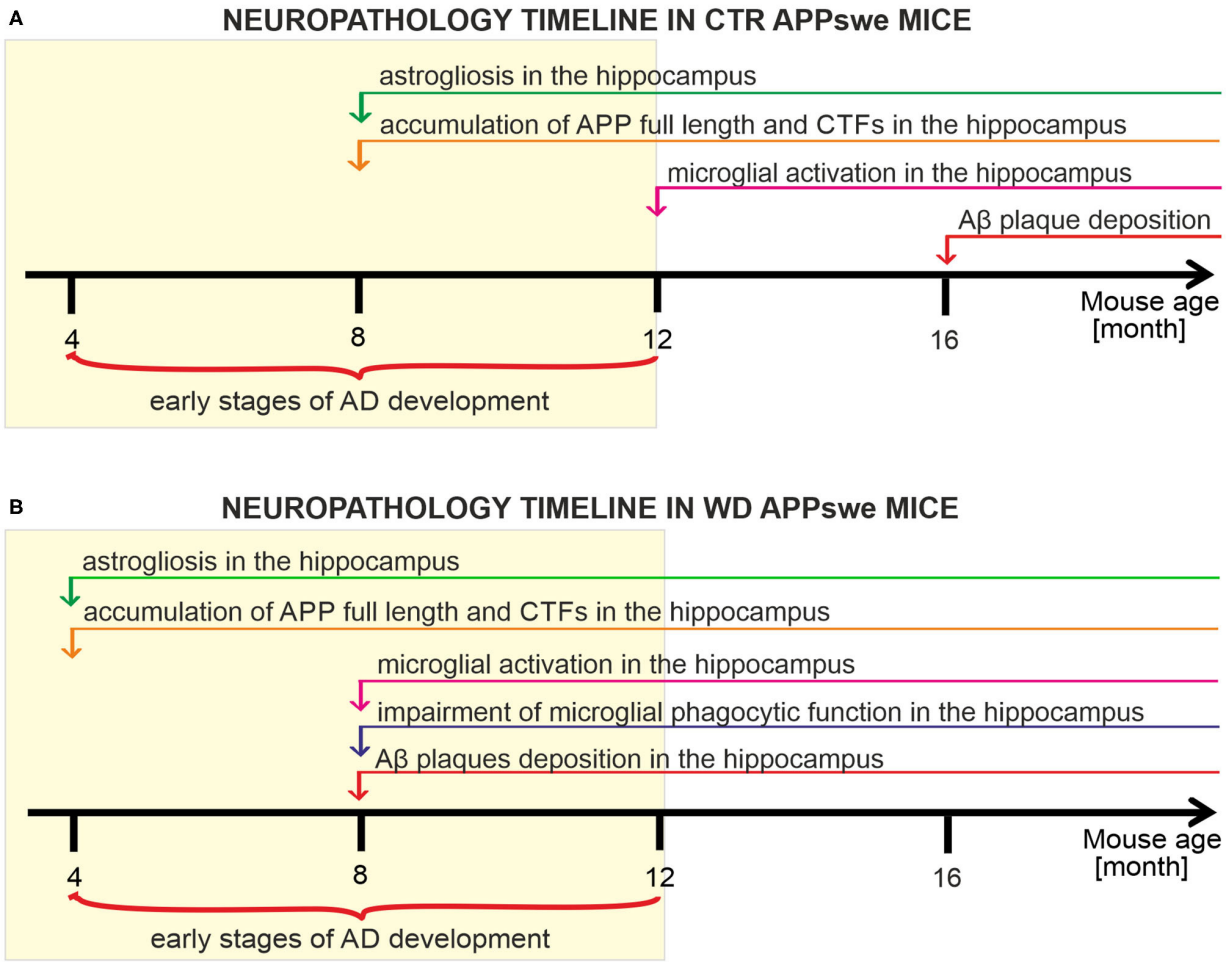
Timelines presenting the neuropathology cascades of events in APPswe mice fed with standard diet compared to mice fed western diet. **(A)** Timeline showing the neuropathological cascades of events in the brain of control APPswe mice fed with standard chow diet. **(B)** Timeline of the WD-induced neuropathological cascades of events in APPswe mouse brain shows the accelerated pathology induced by Western diet feeding.

We have analyzed and characterized the sequence of neuropathological events induced both by the presence of human APP transgene carrying Swedish mutation (Figure 8A), and the presence of human APPswe transgene in mice chronically fed with Western diet (Figure 8B). We observed that this transgene disturbs glial cells' activity revealed by considerable hippocampal astrogliosis in 8-month-old mice and microglial activation in 12M mice. Further, we observed that the mutant APP induces strong enhancement of full-length APP level and cleavage of its C-terminal forms in 8M mice. Despite these processes we observed deposits of Aβ plaques only in 16M APP mice (Figure 8A). We observed that western diet feeding accelerates pathology induced by the APP mutation. Experimental groups of APPswe mice fed with WD developed neuroinflammation and Aβ pathology much earlier than control, untreated, APPswe mice. WD induced astrogliosis after only 3 weeks of WD feeding in the youngest analyzed group of 4M mice, so 4 months earlier than in controls. Moreover, the data indicate activation of pro-inflammatory microglial phenotype with impairment of their phagocytic abilities after 5 months of WD feeding in 8M mice, 4 months earlier than in control mice. Enhancement of full length APP quantity and cleavage of its CTFs was observed after 3 weeks of feeding, in 4M mice, 4 months earlier than in control mice, and accumulation of Aβ plaques after 5 months of feeding in 8M mice, 8 months earlier than in control mice.

These results demonstrate that WD strongly influences, accelerates, and enhances the AD pathological processes induced by the APP mutation in the APPswe mouse model. The most significant novel finding in our study is characterization, for the first time, of the chronology of WD-induced systemic metabolic alterations followed by neuroinflammatory changes, where activation of astroglia preceded microglial activation and brain Aβ pathology.

## Discussion

This study showed the sequence of processes occurring in the body under the influence of WD, leading to the acceleration of the onset of Aβ accumulation in the brain and the progression of Alzheimer's disease. The age groups of APPswe mice analyzed in this study (4M, 8M, 12M, 16M, and 20M) reflect the following age ranges in humans: 17–20, 35, 50, 65–68, 85 years of age, respectively. As shown in this study, the APPswe control (CTR) mice from 4M to 12M correspond to the early pre-plaque stages of AD, without Aβ deposits in the brain in which we observed different pathological processes known to lead to Aβ aggregation. Thus, the APPswe model employed in this study allows for fairly precise translation of the disease timeline to progressive AD stages in human. Furthermore, we found that in this APPswe model, WD induced metabolic disorders and accelerated the onset of pathological changes in the brain including development of amyloidopathy. The oldest APPswe mice maintained on WD for more than 16 months in 20M WD + LPS group showed characteristic features of the late advanced stage of AD development with strongly developed neuropathological lesions. These observations indicate that the WD-treated APPswe mouse model developed by us corresponds well to human AD pathology. The findings presented in this study are also in line with results showing that WD can impair cognition, learning and memory, and enhance or induce pathological features in the brain, both in rodents (Kanoski and Davidson, 2011; Leigh and Morris, 2020) and in humans (Kalmijn et al., 2004; Francis and Stevenson, 2011; Baym et al., 2014; Attuquayefio et al., 2017).

Numerous studies have shown the influence of different western-type diets on metabolic disorders and several articles demonstrated WD-induced upregulation of neuroinflammatory markers in wild-type animals (Thirumangalakudi et al., 2008; Jena et al., 2018; Rutkowsky et al., 2018). These data support the testing of the WD role in AD development in wild-type animals. Our results also provide a strong basis for future research in wild-type mouse models with no AD mutations in the genome, which will allow us to determine whether the western type of nourishment could be a direct cause of the development of SAD as well.

**Figure 9:**
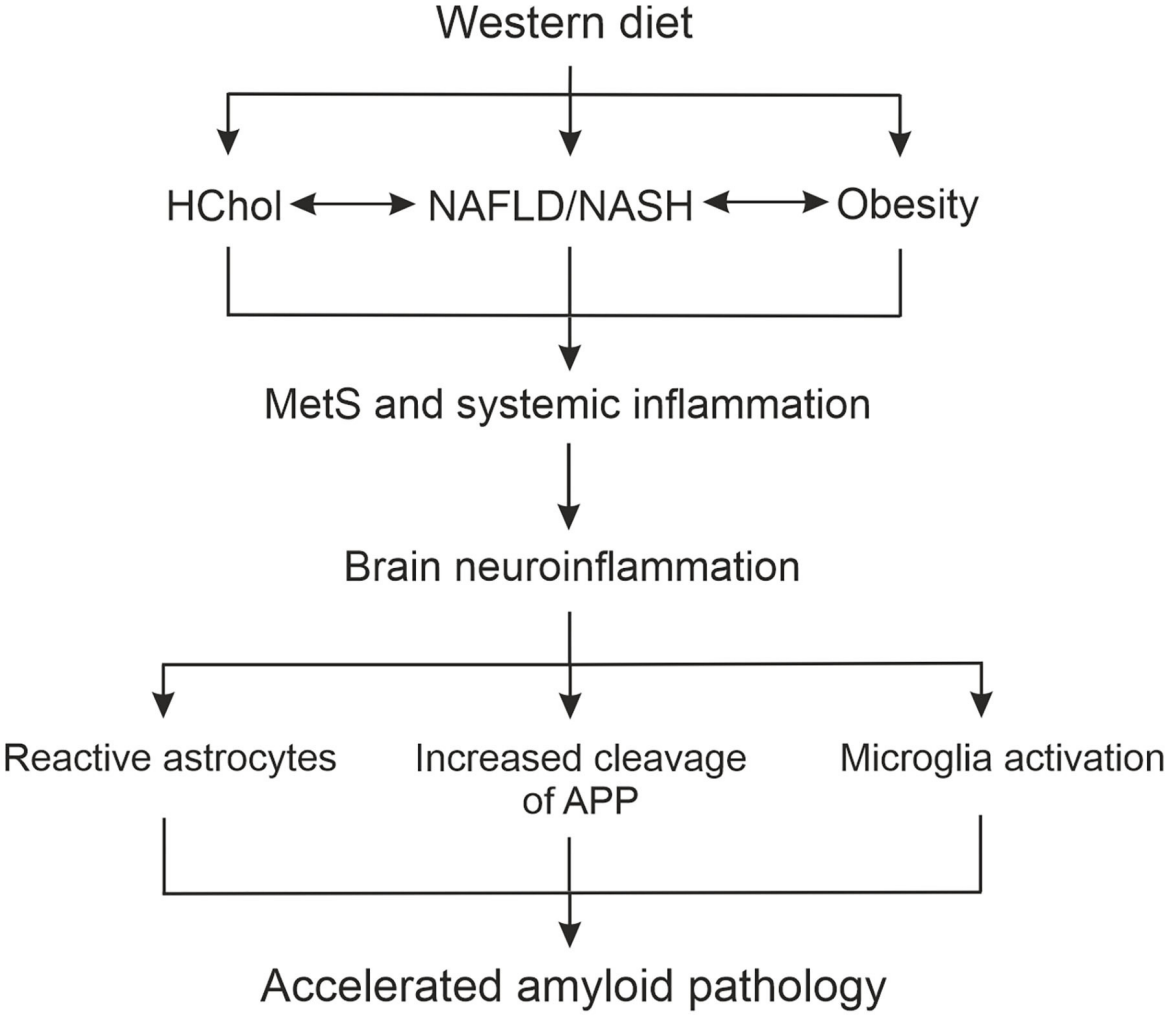
Scheme summarizing the role of liver function in the relationship between WD-induced metabolic and systemic disturbances, and neuropathological changes in the brain leading to accelerated AD development. APP, amyloid precursor protein; HChol, hypercholesterolemia; NAFLD, non-alcoholic fatty liver disease; NASH, non-alcoholic steatohepatitis; MetS, metabolic syndrome.

Obtained results provide support for AD as a systemic disease, not confined to the brain, and for the hypothesis presented schematically in Figure 9 how WD-related metabolic impairment can accelerate brain malfunctioning. In the APPswe mice on WD we found the simultaneous presence of HChol, obesity, NAFLD, and some signs of low-grade inflammation which altogether represent MetS. These peripheral changes induced by WD were associated with the activation of brain glial cells indicative of development of neuroinflammation (Figure 9). Importantly, we observed neuroinflammation already in the brains of the youngest mice, after 3 weeks of WD, at the same time when metabolic disturbances in the periphery became evident. This observation indicates that WD must have caused an almost immediate impairment of BBB brain protection, leading to the activation of glial cells. This then contributed to the intensification of the amyloidogenic APP cleavage path, the formation of new Aβ peptides and at the same time causing impairment in Aβ clearance from the brain (Figure 9). The obtained results confirm the hypothesis that WD feeding acts as a trigger for induction of MetS and disturbances in the activity of innate and adaptive immune systems. Below we discuss in more detail how these processes most probably cross-talk and influence themselves leading to the strong disbalance in peripheral homeostasis and to disruption of the BBB, and later enhance neuroinflammation and Aβ deposition in the hippocampus.

### WD-Induced Hypercholesterolemia in AD Development

Within 3 weeks of WD feeding we observed an increase in body weight and hypercholesterolemia (HChol), defined as increased levels of LDL (“bad cholesterol”) and low levels of HDL (“good cholesterol”) in blood plasma. Our results are in line with HChol as a known risk for AD and other neurodegenerative diseases (Kosari et al., 2012; Xue-Shan et al., 2016). In particular, our data support the observation that midlife HChol is usually associated with earlier AD onset in human (De Oliveira et al., 2014), particularly regarding higher levels of LDL inducing cortical amyloid deposition (Rodríguez et al., 2006). Brain cholesterol is one of the crucial elements for maintaining brain homeostasis and neuronal functions (Dietschy and Turley, 2001; Vance, 2012), building up the myelin sheaths of oligodendrocytes, astrocytes and neuronal axons (Mahley and Rall, 2000; Puglielli et al., 2003; Canevari and Clark, 2007). Under normal conditions, an efficiently functioning BBB effectively counteracts the influx of peripheral cholesterol into the brain, but under the influence of metabolic disorders and associated inflammatory factors BBB permeability is enhanced (Chakraborty et al., 2017). Cholesterol can pass through the BBB in both directions, and its final serum level is an outcome of these processes (Umeda et al., 2012).

High levels of diet-derived serum cholesterol cause its conversion into 27-hydroxycholesterol, the cholesterol form able to flow freely through the BBB. It is mediated by enhanced levels of cytochrome P450 CYP27A1 and CYP7A1 isoforms in hepatocytes during NAFLD (Testa et al., 2016; Zhang et al., 2018; Gamba et al., 2019; Chen et al., 2020). The increased influx of 27-hydroxycholesterol into the brain contributes to the induction of brain oxidative stress and development of AD (Testa et al., 2016; Ioannou et al., 2017; Mast et al., 2017; Loera-Valencia et al., 2019; Chew et al., 2020; Wong et al., 2020). The pathological consequences of brain HChol are also linked with cholesterol function as an important component of cell membrane lipid rafts, where APP and β- and γ-secretases involved in its amyloidogenic proteolysis are located. Evidence shows that HChol and an increased brain cholesterol level are connected with increased cholesterol concentration in membrane lipid rafts which enhances APP amyloidogenesis and Aβ production in neurons (Allinquant et al., 2014) and astrocytes (Xiu et al., 2006; Avila-Muñoz and Arias, 2015).

### WD-Induced Obesity in AD Development

Our results in WD-fed mice are also in agreement with extensive human data demonstrating a clear link between WD-induced obesity and the onset of AD (Solfrizzi et al., 2004; Whitmer et al., 2008; Gustafson et al., 2009, 2012; Besser et al., 2014) with obesity increasing the chance of AD development 6-fold (Kivipelto et al., 2005). Mechanistically, storage capacities of obese adipose tissue are exceeded and, in such conditions, free fatty acids “spill over” and accumulate in metabolic tissues, such as skeletal muscle, liver and pancreas (van Herpen and Schrauwen-Hinderling, 2008). Excessive exposure of adipocytes and adipocyte-tissue-resident immune cells to free fatty acids initiates pro-inflammatory signaling pathways (Lyons et al., 2016) resulting in the secretion of pro-inflammatory agents from adipocytes and resident immune cells leading to lipotoxicity and insulin resistance (Hotamisligil, 2017; Burhans et al., 2019). In line with the described factors contributing to low-grade inflammation, our data also indicated signs of a systemic low-grade inflammatory state. Hematological analysis of blood demonstrated increases in the level of WBC, especially GRA and MON, after only 3 weeks of WD feeding, and in LYM after 5 months of WD, and shifts of the analyzed parameters to the upper limits of physiological norms. It indicates an almost immediate WD-induced activation of the entire arsenal of immune responses, both innate as well as adaptive, to defend against the damaging effects of unhealthy dietary components.

In the group of 8M animals (WD and WD + LPS) a noticeable increase both in WBC and in body weight was observed. The correlation between WBC count and body weight is known to be caused by leptin released from adipose tissue, which stimulates hematopoiesis (Wilson et al., 1997). The presence of leptin receptors on hematopoietic stem cells (both in mice and humans), and the observation that leptin stimulates myelopoiesis and lymphopoiesis further indicates the link between adipose tissue and WBC count (Laharrague et al., 2000). Moreover, given that high-fat diet in mice is known to cause an increase in neutrophil recruitment from blood into adipose tissue (Elgazar-Carmon et al., 2008) it seems plausible that neutrophils play a role in initiating the inflammatory cascade in response to obesity. Adipose tissue neutrophils produce chemokines and cytokines, facilitating macrophage infiltration, which could contribute specifically to the diet-derived chronic low-grade inflammation.

A decrease in body weight detected in the WD-fed group at 12 months of age can be explained by a loss of weight due to the appearance of AD symptoms and development of serious metabolic disorders probably resulting in decreased food intake. In addition, the high cholesterol content of WD in this study may represent another factor contributing to body weight loss in long-fed animals, given that a high-cholesterol diet in the 3xTg-AD model caused unexpected weight loss in mice after 12 months of feeding (Hohsfield et al., 2014). It seems that AD-related transgenes and associated genotypes may modify cholesterol metabolism in the periphery and its subsequent accumulation in fatty tissues resulting in elevated stress and anxiety, culminating in body weight loss.

### WD-Induced Liver Pathology in AD Development

Among organs responsive to pro-inflammatory factors is the liver. The immune response in the liver plays a pivotal role in the development of NAFLD, but the precise regulation of the immune response is unclear and liver neutrophil and macrophage activation is considered to be a double-edged sword (Xu et al., 2014). In accordance with this knowledge, we observed in our study an increase in the level of granulocytes and monocytes in the blood during WD feeding, alongside increased infiltration of pro-inflammatory cells into the liver tissue in the course of NASH. We have shown that only 3 weeks of feeding WD to mice caused hepatocyte hypertrophy and lipid vacuoles to form within. These morphological changes in liver tissue worsen with age and with continued WD feeding, finally leading to hepatocellular ballooning and macrovesicular steatosis, which are hallmarks of developing NASH in older mice (8M, 12M). The advanced WD-derived damage in the liver together with the accompanying infiltration of inflammatory cells confirms the development of NAFLD.

Our results are consistent with the knowledge that typical WD ingredients are responsible for such liver impairment (Berná and Romero-Gomez, 2020). Also, WD modulates the accumulation of triglycerides and antioxidants in the liver, which affects insulin sensitivity and post-prandial triglyceride metabolism (Musso et al., 2003). Saturated fatty acids (SFAs) in WD significantly increase liver steatosis because of the induction of lipogenesis in the liver and lipolysis in adipose tissue. Moreover, SFAs affect glutathione metabolism leading to oxidative stress (Franko et al., 2018; Rosqvist et al., 2019). Monosaccharides and disaccharides abundant in WD-type meals also affect liver functioning. There is a strong association between the risk of NAFLD and products containing high amounts of sucrose or fructose like corn syrup, cakes, soft drinks and sugary snacks. Like SFAs, fructose from food stimulates lipogenesis in the liver and leads to steatosis, but also manifests with increased levels of plasma alanine aminotransferases, an indicator of functional liver impairment (Chiu et al., 2018; Berná and Romero-Gomez, 2020).

The physiological role of the liver involves many processes that are involved in mechanisms related to the development and progression of AD. Structural and/or functional damage of the liver reduces its ability to effectively remove and degrade Aβ. In patients with liver diseases, low hepatic expression of LRP1 and high levels of circulating Aβ are observed because Aβ clearance decreases due to low hepatic LRP1 activity (Wang et al., 2017). A recent epidemiological study demonstrated that comorbidities associated with mild cognitive impairment and dementia due to AD were predominantly diet-induced disorders, such as NAFLD, cirrhosis, cerebrovascular diseases, or type 2 diabetes mellitus all of which are characterized by a reduction in removal of Aβ (Bassendine et al., 2020). NAFLD is strongly related to diet-induced hepatic insulin resistance (Kumashiro et al., 2011). In healthy conditions insulin promotes LRP1 translocation to the cell membrane in hepatocytes and favoring Aβ clearance by LRP1, but insulin resistance makes this process poor or impossible leading to a general increase of Aβ level (Tamaki et al., 2007).

Our results also showed that liver damage is correlated with the deposition of Aβ in the brain. WD accelerated the accumulation and deposition of Aβ by about 8 months compared to control APPswe mice. Furthermore, liver pathology, such as NAFLD and NASH enhances the level of cholesterol in the bloodstream, particularly of 27-hydroxycholesterol, the form able to flow freely through the BBB. This accumulation of cholesterol in the brain favors production of Aβ in lipid rafts as described earlier, contributing to the vicious circle to AD progression.

### WD-Derived BBB Disruption as an “Open Gate” Into the Brain in AD Progression

BBB disruption seems to be a very early disorder occurring simultaneously with the activation of an immune response to damaging dietary factors. In particular, Aβ level in the brain depends on the continuous control of Aβ influx and efflux via receptors RAGE and LRP1 located at the BBB (Zlokovic et al., 2010). Intake of diets high in SFAs, cholesterol and carbohydrates increases levels of circulating Aβ, disturbs its peripheral clearance by the liver and impairs brain Aβ clearance and efflux by decreased LRP1 and increased RAGE protein expression or activity (Kim et al., 2016; Gali et al., 2019). In turn, BBB dysfunction triggers oxidative stress and neuroinflammation, which causes enhancement of the activity of β-secretase and γ-secretase promoting generation of a new portion of Aβ peptides (Cai et al., 2018).

Since clinical manifestations of AD start from impairment of memory, we analyzed WD-dependent alteration in the brain predominantly in the hippocampus. In addition, the hippocampus is especially sensitive to changes in nutrients supplied to the body with food, as well as to circulating toxins and metabolic products. WD affects predominantly the CA1 and CA3 areas, subiculum and dentate gyrus (Hargrave et al., 2016). The large pyramidal neurons within the hippocampus epitomize the high metabolic demand and have a unique metabolic profile that makes these neurons especially sensitive to damage from environmental and metabolic insults. The hippocampus is particularly susceptible to high levels of SFAs and simple sugars (Kanoski and Davidson, 2010; Hsu and Kanoski, 2014). Moreover, the consumption of WD reduces the level of BDNF in the hippocampus (Molteni et al., 2002), increases neuroinflammation (Pistell et al., 2010), impairs synaptic plasticity (Stranahan et al., 2008), and alters dendritic morphology (Granholm et al., 2008), glutamatergic signaling via upregulation of synaptic clearance mechanisms leading to NMDA receptor desensitization (Valladolid-Acebes et al., 2012) and blood vessel structure (Freeman et al., 2011).

### WD-Induced Activation of Astrocytes in AD Progression

This study showed that activation of astrocytes in WD-fed mice occurred relatively early, together with HChol, NAFLD, and enhanced brain accumulation of APP and its cleavage to CTFs, and preceded microglial activation. In agreement with these data, studies on human brain samples showed an increase in reactive gliosis that appears to precede the development of the characteristic lesions of AD (Wharton et al., 2009; Sidoryk-Wegrzynowicz et al., 2011).

The astrocyte activation was probably induced as a common result of HChol and NAFLD. It was shown earlier, that *in vitro* cholesterol exposure induced astrocyte activation, increased APP content, and enhanced APP-BACE-1 interaction. These effects were associated with an enrichment of lipid rafts' cholesterol patches in the astrocyte membrane and with increased reactive oxygen species production, that results in astrocytes activation (Avila-Muñoz and Arias, 2015). In turn, enlarged lipid rafts harboring activated receptors and adaptor molecules serve as an organizing platform to initiate inflammatory signaling (Miller et al., 2020).

Astrocyte activation leads to changes in both expression of aquaporin 4 and its redistribution from astrocytic end-feet membranes to non-end-feet ones in BBB parenchyma and plays an important role in the proper functioning of the lymphatic system responsible for brain clearance of accumulated toxic Aβ, which results in enhanced brain Aβ accumulation (Yang et al., 2011; Iliff et al., 2014; Kress et al., 2014; Xu et al., 2015; Peng et al., 2016; Zhang et al., 2017). Also, activated astrocytes produce and release pro-inflammatory molecules that may also be critical for the generation of Aβ (Avila-Muñoz and Arias, 2014).

### WD-Induced Microglial Impairment in AD Progression

Extensive evidence suggests that microglia play a significant role in development of AD. Microglial phagocytosis in the early stage of AD was considered beneficial, being the key mechanism preventing formation, and promoting removal, of Aβ plaques (Salter and Stevens, 2017; Richter et al., 2020). However, during disease progression, microglial phagocytosis seems to become insufficient and rather detrimental, combined with their pro-inflammatory activity, not counteracting but promoting pathology (Vogels et al., 2019; Streit et al., 2020). In agreement with this view, our study revealed the accelerated activation of microglia and its M1 pro-inflammatory polarization profile after 5 months of WD in 8M APPswe mice. We observed microglial activation and changes in their polarization profile 4 months later than changes in diet-induced astrocyte activation, which suggests that microglia disturbances can be triggered in part by the astrocyte inflammatory response.

WD-induced hippocampal surplus of Iba1 may display a pro-inflammatory profile revealed by both activated brain resident microglia and pro-inflammatory monocyte-derived macrophages (MDMs) infiltrating the brain from the periphery through the disrupted BBB. This possibility is consistent with the observed enhancement of the number of monocytes in the blood in 4M and 8M APPswe mice fed with WD from 3 weeks to 5 months and its normalization to basal levels in 12M mice; it suggests brain infiltration by monocytes at the earlier stages, and indicates the possible role of peripheral immune cells like MDMs in induction of impairment of microglial function and neuroinflammation.

Some data underscore the crucial role of diet-derived SFAs in microglial activation and neuroinflammation (Pistell et al., 2010; Gupta et al., 2012; Wang et al., 2012). Given that SFAs (e.g., palmitic and stearic acids) are free to cross the BBB, especially under HFD conditions (Niu et al., 2016), brain SFA homeostasis is dependent on SFA levels in the periphery. It is further conceivable that diets rich in SFAs may increase brain uptake of SFAs from plasma through the BBB (Wang et al., 1994) resulting in their brain accumulation. WD-derived SFAs in the brain can impair microglial function via signaling involving microglial CD14-TLR4-MD2 complex which induces NFκB signaling, triggering secretion of pro-inflammatory cytokines and later neuroinflammation (Wang et al., 2012; Rocha et al., 2016; Moser et al., 2018).

This study also showed impairment in microglial phagocytosis abilities, revealed by hippocampal decrease of CD68, which correlated with the initiation of Aβ plaque formation. These observations suggest that microglial polarization to the M1 pro-inflammatory state leads to the loss of their abilities to clear Aβ debris resulting in Aβ accumulation, amyloid plaque deposition, and deepening of neuroinflammation.

### Differences in Responses to WD and LPS

Our study focused on comparison of WD-fed animals to the normal-diet-fed group. Treatment with LPS was analyzed additionally as a control, and we employed statistical methodology accordingly. Moreover, we did not analyze the age factor but we focused on the impact of a given treatment in groups at the same age, where each treatment was considered independently. Nevertheless, the study provided some data for comparison of LPS-induced changes to WD-induced changes during AD development.

Numerous bacterial molecules, including LPS, activate the immune response by interaction with pattern recognition receptors, such as TLRs. LPS particles are bound to the lipopolysaccharide binding protein (LBP) and then attached to CD14 protein present on the surface of macrophages, monocytes, granulocytes, and B lymphocytes, which causes an increase in integrin expression and release of large amounts of pro-inflammatory cytokines (IL-1,−6, TNFα) (Wright et al., 1989; Zinöcker and Lindseth, 2018). This results in the activation of endothelial cells and transmits the signal to produce acute-phase proteins in the liver (Moshage, 1997).

On the other hand, the triggers of the inflammatory reaction in obesity caused by WD are not quite clear and the literature provides many hypotheses. The activation of an obesity-related innate immune response is likely to involve pattern recognition receptors including TLRs (Monteiro and Azevedo, 2010).

There was a significant increase in mortality in the WD group relative to LPS-induced WD mice. This was most likely due to “saturation” of TLR receptors by palmitates from food, which prevents activation of the LPS-dependent pathway characterized by acute inflammation (Milanski et al., 2009). It seems that aged WD mice had significantly higher levels of inflammation than WD + LPS, which is correlated with an increase in pro-inflammatory cytokine synthesis. That is supported by studies in which cytokine synthesis (e.g., IL-6 and TNF) before and after LPS stimulation was measured in animals fed with WD. The animals presented increased transcription of cytokines before stimulation and a significant decrease in their expression afterward (Napier et al., 2019).

Another possible, complementary, mechanism to increase 16M WD rodent group mortality compared to 16M WD + LPS may be the effect of dietary components on the HPA axis. Studies on the effects of short- and long-term WD on hypothalamic and hippocampal inflammatory reactions in mice exposed to acute LPS-induced inflammation have shown that animals on a HFD diet have a significant increase in the expression of glucocorticoid receptor of the hypothalamus after LPS stimulation compared to WD mice in which this expression is markedly lower (De Souza et al., 2005; Milanski et al., 2009; Thaler et al., 2012; Pohl et al., 2014; Astiz et al., 2017). Glucocorticoids act in negative feedback loops to suppress the HPA-axis, creating a shift from pro-inflammatory to anti-inflammatory immune responses (Elenkov and Chrousos, 1999). It can therefore be presumed that the LPS combined with WD had, in a way, a protective function to suppress the pro-inflammatory response in the central nervous system by stimulating peripheral corticosterone production (Elenkov and Chrousos, 1999; John and Buckingham, 2003).

### Conclusions

The comparison of the described results with the literature indicates a good reflection of the animal model used both in terms of disease progression over time and the observed systemic changes caused by WD and LPS. The neuropathological pattern of Aβ platelets obtained in animals does not differ from that observed in humans.

This study showed that the westernized pattern of nourishment: (1) leads to development of metabolic syndrome including HChol, obesity, NAFLD and signs of low-grade systemic inflammation, (2) induces the neuroinflammatory processes revealed by glial cell hyperactivation, and finally (3) accelerates amyloid pathology in the AD brain. This indicates that such diet-derived metabolic alterations as hypercholesterolemia and obesity, cause systemic low-grade inflammation and NAFLD, which is strongly linked to the impairment of liver-BBB-brain axis. BBB malfunctioning and liver disruption accelerate neuroinflammatory processes revealed by glial cell over-activity and deposition of Aβ plaques in the brain.

Analysis of these results in the light of existing literature indicates that AD is a disorder not only of the brain but of the whole organism. Peripheral processes and organs can play a crucial role in AD development. In particular, diet-induced liver damage leads to a failure in Aβ degradation and clearance that seems to be one of the main factors accelerating the development of AD. Thus, the westernized pattern of nourishment should be considered as an important modifiable risk factor of AD development. A healthy, balanced, diet and regular controlling of the metabolic state of the body and prevention and treatment of liver diseases may be some of the most efficient AD prevention methods and would also support treatment of patients diagnosed with AD.

## Data Availability Statement

The raw data supporting the conclusions of this article will be made available by the authors, without undue reservation.

## Ethics Statement

The animal study was reviewed and approved by 1st Local Ethical Committee for Experiments on Animals in Warsaw, Faculty of Biology, University of Warsaw, No. 630/2018.

## Author Contributions

AW-G, AM-P, and UW: conceptualization, funding acquisition, and writing—original draft. AW-G, AM-P, DC, MW, and JD: conduct of experiments. AW-G, AM-P, DC, and UW: results analysis and interpretation. AW-G, AM-P, UW, DC, and MW: writing—review and editing. All authors have read and agreed to the submitted version of the manuscript.

## Conflict of Interest

The authors declare that the research was conducted in the absence of any commercial or financial relationships that could be construed as a potential conflict of interest.

## Abbreviations

4M: 4-month-old
8M: 8-month-old
12M: 12-month-old
16M: 16-month-old
20M: 20-month-old
APP: amyloid precursor protein
CTFs: C-terminal end fragments
GRA: granulocytes
HChol: hypercholesterolemia
LYM: lymphocytes
MetS: metabolic syndrome
MON: monocytes
NAFLD: non-alcoholic fatty liver disease
NASH: non-alcoholic steatohepatitis
SFAs: saturated fatty acids
WBC: white blood cells
WD: western diet.
