# Supplementary material for: Western Diet Induces Impairment of Liver-Brain Axis Accelerating Neuroinflammation and Amyloid Pathology in Alzheimer's Disease
Source: Front Aging Neurosci. 2021 Apr 1;13:654509. doi: 10.3389/fnagi.2021.654509 (PMC8046915; doi:10.3389/fnagi.2021.654509)
Supplement: Supplementary file 1 [file Table_1.DOCX]

***Supplementary Material***

# Supplementary Tables and Figures

**1.1 Supplementary Tables**

**Table S1.** Results from multiple comparison post-hoc tests

| **Figure 1A: Cholesterol** | | | |
| --- | --- | --- | --- |
| **Age group: post-hoc test** | **Comparison of experimental groups** | **Significance** | **p-value** |
| 4M: Bonferroni's post-hoc | CTR vs. LPS | ns | >0.999 |
|  | CTR vs. WD | *** | **<0.001** |
|  | CTR vs. WD+LPS | *** | **<0.001** |
|  | LPS vs. WD | *** | **<0.001** |
|  | LPS vs. WD+LPS | *** | **<0.001** |
|  | WD vs. WD+LPS | ns | >0.999 |
| 8M: Bonferroni's post-hoc | CTR vs. LPS | ns | >0.999 |
|  | CTR vs. WD | *** | **<0.001** |
|  | CTR vs. WD+LPS | *** | **<0.001** |
|  | LPS vs. WD | *** | **<0.001** |
|  | LPS vs. WD+LPS | *** | **<0.001** |
|  | WD vs. WD+LPS | ns | 0.215 |
| 12M: Bonferroni's post-hoc | CTR vs. LPS | ns | >0.999 |
|  | CTR vs. WD | ns | 0.602 |
|  | CTR vs. WD+LPS | ** | **0.009** |
|  | LPS vs. WD | ns | 0.524 |
|  | LPS vs. WD+LPS | * | **0.011** |
|  | WD vs. WD+LPS | ns | 0.603 |
| **Figure 1B: Body weight** | | | |
| **Age group: post-hoc test** | **Comparison of experimental groups** | **Significance** | **p-value** |
| 4M: Bonferroni's post-hoc | CTR vs. LPS | ns | >0.999 |
|  | CTR vs. WD | ns | 0.811 |
|  | CTR vs. WD+LPS | ns | 0.078 |
|  | LPS vs. WD | ns | 0.225 |
|  | LPS vs. WD+LPS | * | **0.024** |
|  | WD vs. WD+LPS | ns | >0.999 |
| 8M: Bonferroni's post-hoc | CTR vs. LPS | ns | >0.999 |
|  | CTR vs. WD | *** | **<0.001** |
|  | CTR vs. WD+LPS | ns | 0.323 |
|  | LPS vs. WD | *** | **<0.001** |
|  | LPS vs. WD+LPS | ns | 0.344 |
|  | WD vs. WD+LPS | *** | **<0.001** |
| **Figure 3B: MON** | | | |
| **Age group: post-hoc test** | **Comparison of experimental groups** | **Significance** | **p-value** |
| 8M: Dunn's post-hoc + Bonferroni's correction | CTR vs. WD | ns | 0.628 |
|  | CTR vs. WD+LPS | * | **0.031** |
|  | WD vs. WD+LPS | ns | 0.469 |
| **Figure 4A: GFAP** | | | |
| **Age group: post-hoc test** | **Comparison of experimental groups** | **Significance** | **p-value** |
| 4M: Bonferroni's post-hoc | CTR vs. LPS | *** | **<0.001** |
|  | CTR vs. WD | *** | **<0.001** |
|  | CTR vs. WD+LPS | ** | **0.002** |
|  | LPS vs. WD | ns | >0.999 |
|  | LPS vs. WD+LPS | ns | >0.999 |
|  | WD vs. WD+LPS | ns | >0.999 |
| **Figure 5A: Iba1** | | | |
| **Age group: post-hoc test** | **Comparison of experimental groups** | **Significance** | **p-value** |
| 4M: Dunn's post-hoc + Bonferroni's correction | CTR vs. LPS | ns | 0.108 |
|  | CTR vs. WD | ns | >0.999 |
|  | CTR vs. WD+LPS | ns | 0.258 |
|  | LPS vs. WD | ** | **0.002** |
|  | LPS vs. WD+LPS | *** | **<0.001** |
|  | WD vs. WD+LPS | ns | >0.999 |
| 8M: Dunn's post-hoc + Bonferroni's correction | CTR vs. LPS | ns | >0.999 |
|  | CTR vs. WD | * | **0.018** |
|  | CTR vs. WD+LPS | *** | **<0.001** |
|  | LPS vs. WD | ns | 0.479 |
|  | LPS vs. WD+LPS | ******* | **<0.001** |
|  | WD vs. WD+LPS | ns | 0.053 |
| 12M: Dunn's post-hoc + Bonferroni's correction | CTR vs. LPS | ns | >0.999 |
|  | CTR vs. WD | * | **0.012** |
|  | CTR vs. WD+LPS | * | **0.012** |
|  | LPS vs. WD | * | **0.012** |
|  | LPS vs. WD+LPS | * | **0.013** |
|  | WD vs. WD+LPS | ns | >0.999 |
| **Figure 5B: P2RY12** | | | |
| **Age group: post-hoc test** | **Comparison of experimental groups** | **Significance** | **p-value** |
| 8M: Dunn's post-hoc + Bonferroni's correction | CTR vs. LPS | ns | 0.701 |
|  | CTR vs. WD | ns | 0.201 |
|  | CTR vs. WD+LPS | ns | 0.061 |
|  | LPS vs. WD | ns | >0.999 |
|  | LPS vs. WD+LPS | ns | >0.999 |
|  | WD vs. WD+LPS | ns | >0.999 |
| 12M: Dunn's post-hoc + Bonferroni's correction | CTR vs. LPS | ns | 0.061 |
|  | CTR vs. WD | ns | 0.125 |
|  | CTR vs. WD+LPS | ns | >0.999 |
|  | LPS vs. WD | ns | >0.999 |
|  | LPS vs. WD+LPS | ns | 0.300 |
|  | WD vs. WD+LPS | ns | 0.577 |
| Figure 5C: CD68 | | | |
| Age group: post-hoc test | Comparison of experimental groups | Significance | p-value |
| 12M: Dunn's post-hoc + Bonferroni's correction | CTR vs. LPS | ns | 0.554 |
|  | CTR vs. WD | * | **0.013** |
|  | CTR vs. WD+LPS | ns | >0.999 |
|  | LPS vs. WD | ns | >0.999 |
|  | LPS vs. WD+LPS | ns | 0.441 |
|  | WD vs. WD+LPS | ** | **0.009** |
| **Figure 6B: APP full length** | | | |
| **Age group: post-hoc test** | **Comparison of experimental groups** | **Significance** | **p-value** |
| 4M: Bonferroni's post-hoc | CTR vs. LPS | ns | 0.340 |
|  | CTR vs. WD | * | 0.015 |
|  | CTR vs. WD+LPS | * | 0.011 |
|  | LPS vs. WD | ns | >0.999 |
|  | LPS vs. WD+LPS | ns | >0.999 |
|  | WD vs. WD+LPS | ns | >0.999 |
| **Figure 6B: APP CTFs** | | | |
| **Age group: post-hoc test** | **Comparison of experimental groups** | **Significance** | **p-value** |
| 4M: Bonferroni's post-hoc | CTR vs. LPS | ns | 0.971 |
|  | CTR vs. WD | ****** | **0.008** |
|  | CTR vs. WD+LPS | ****** | **0.003** |
|  | LPS vs. WD | ns | 0.298 |
|  | LPS vs. WD+LPS | ns | 0.158 |
|  | WD vs. WD+LPS | ns | >0.999 |

ns - non significant; * p<0.05; ** p<0.01; *** p<0.001;

## 1.2 Supplementary Figures

**
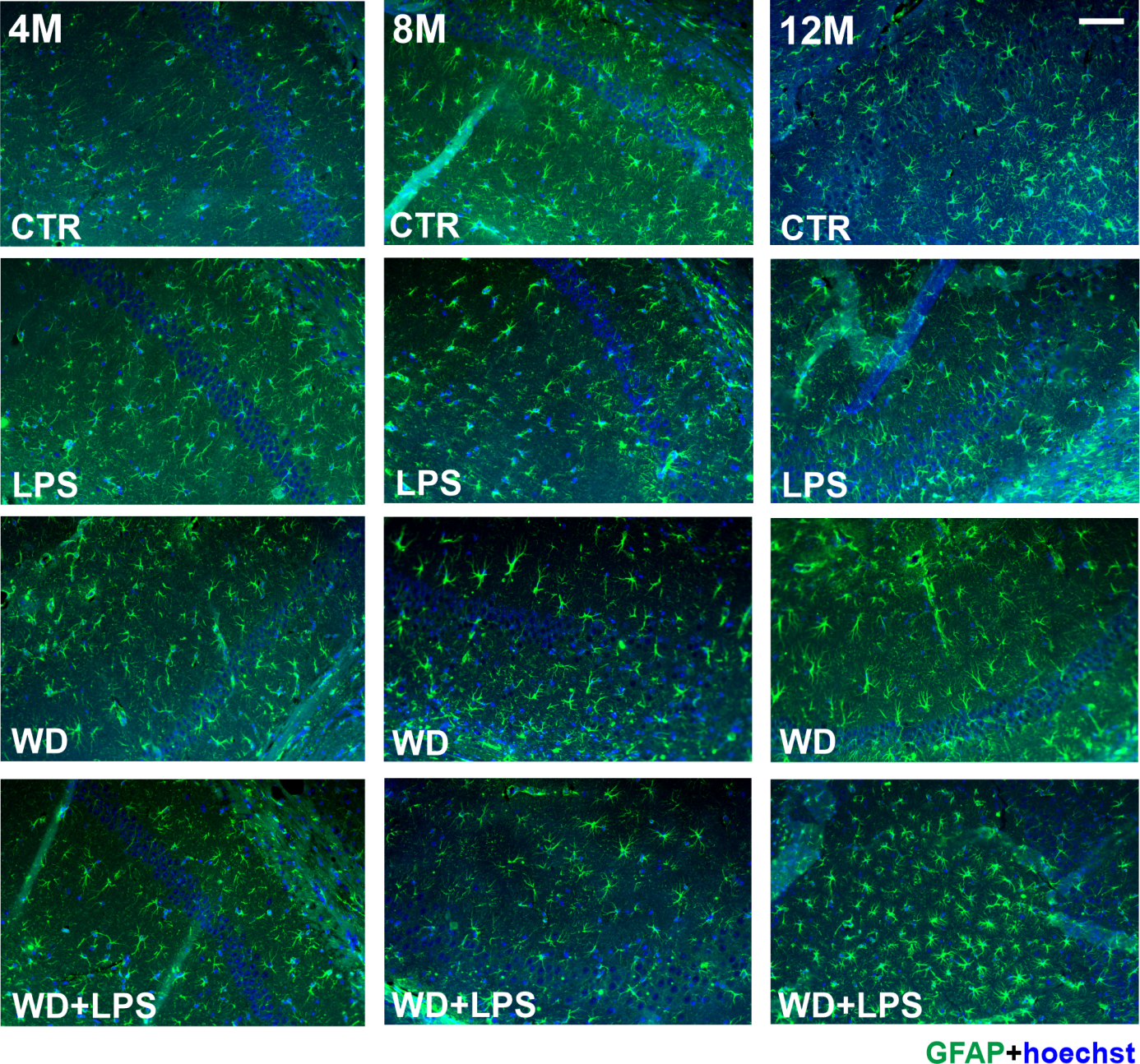
**

**Figure S1.** Comparative qualitative immunofluorescence analysis showing the changes in GFAP staining in CTR, LPS, WD and WD+LPS experimental groups in 4, 8, and 12-months old APPswe mice hippocampus; scale bar = 50µm; magnification x20; green fluorescence - GFAP, blue fluorescence - hoechst (nuclei).


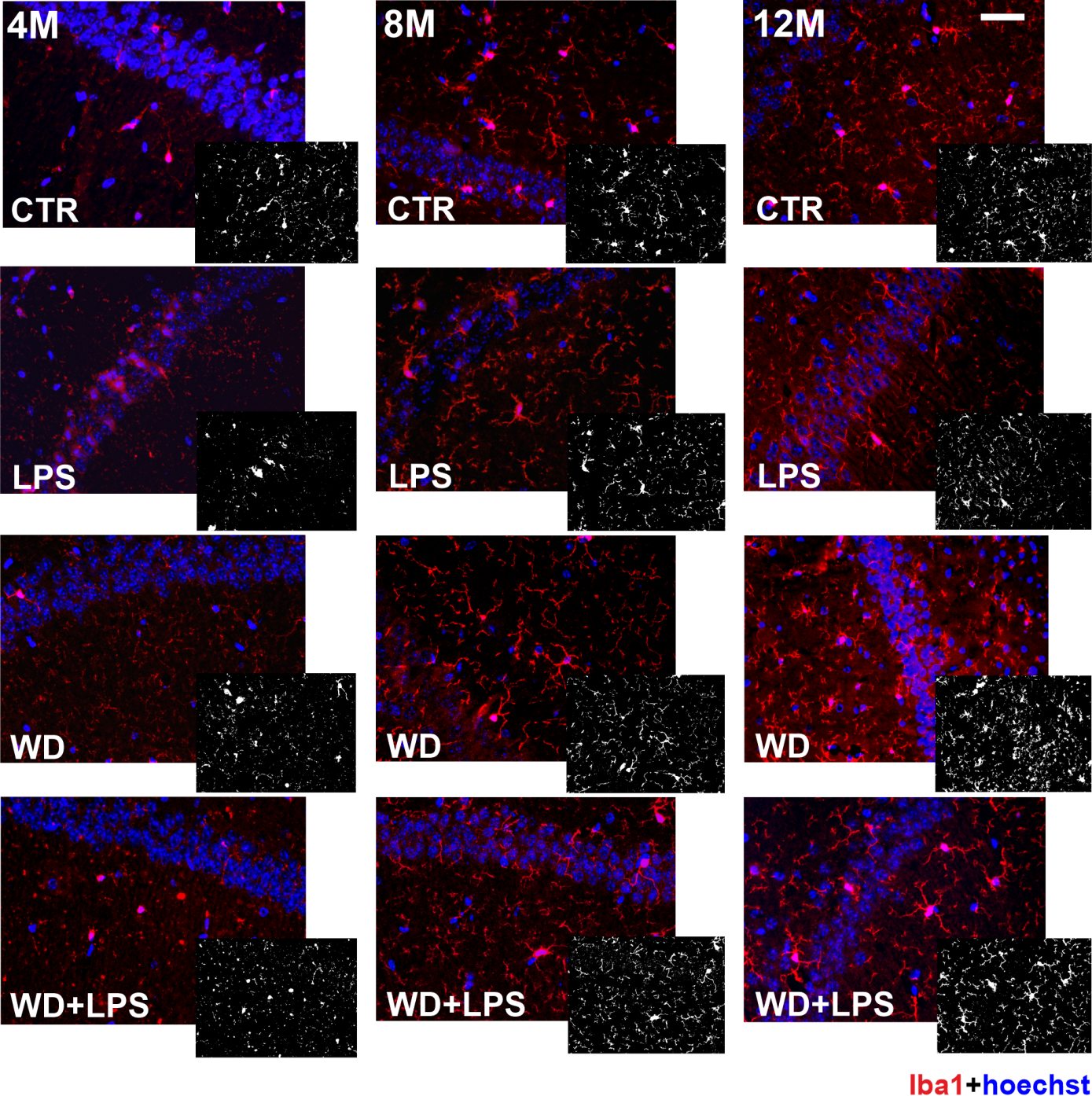


**Figure S2.** Comparative qualitative immunofluorescence analysis showing the changes in Iba1 positive area staining in CTR, LPS, WD and WD+LPS experimental groups in 4, 8, and 12-months old APPswe mice hippocampus; scale bar = 50µm; magnification x20; red fluorescence - Iba1, blue fluorescence - hoechst (nuclei).


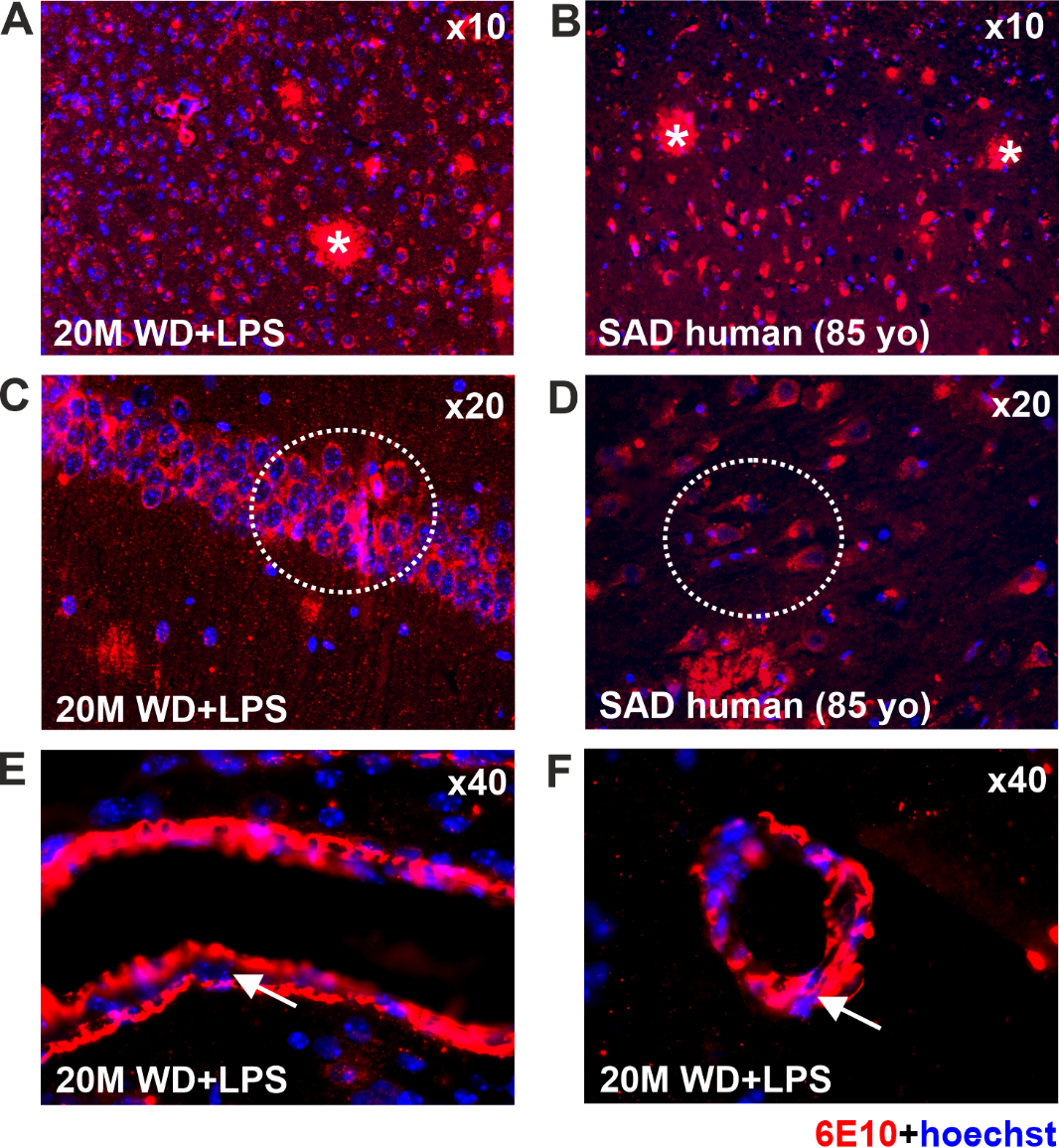


**Figure S3. Similar amyloidopathy in the hippocampus of APPswe mice fed with WD  (WD + LPS group) and in the sporadic AD patient (SAD) indicates that the mouse model is adequate for studying human pathology.**

The microphotographs show immunofluorescence labeling of Aβ (6E10) in the hippocampal tissue of:  **(A, C, E, F)** 20-month-old APP mice from the WD + LPS group (fed with WD and LPS-treated) compared to **(B, D)** hippocampal tissue of a sporadic Alzheimer’s disease (SAD) patient. **(A, B)** - lens magnification x10, **(C, D)** - lens magnification x20, **(E, F)** - lens magnification x40. In **(A, B)** the occurrence of Aβ plaques is marked with *, in **(C, D)** unaggregated Aβ in the cytoplasm of hippocampal neurons is indicated with a dotted line. Additionally, in **(E, F)** cerebral amyloid angiopathy in the mouse brain is indicated with arrows; red fluorescence - 6E10 (Aβ), blue fluorescence - hoechst (nuclei).

The mortality of animals especially in the groups fed with WD was higher than expected, probably due to severe metabolic dysfunctions and occurrence of liver tumors. Due to these reasons it was not possible to keep an adequate number of animals alive until 16 and 20 months of age and these groups are not compared in this figure.
